# Health-Promoting Nature of *Lactococcus lactis* IBB109 and *Lactococcus lactis* IBB417 Strains Exhibiting Proliferation Inhibition and Stimulation of Interleukin-18 Expression in Colorectal Cancer Cells

**DOI:** 10.3389/fmicb.2022.822912

**Published:** 2022-05-25

**Authors:** Przemysław Sałański, Magdalena Kowalczyk, Jacek K. Bardowski, Agnieszka K. Szczepankowska

**Affiliations:** Institute of Biochemistry and Biophysics, Polish Academy of Sciences, Warsaw, Poland

**Keywords:** whole genome sequencing, probiotic bacteria, *Lactococcus lactis*, colorectal cancer, proliferation inhibition, adhesion, interleukin 18

## Abstract

Lactic acid bacteria (LAB) are Gram-positive bacteria which are considered for use as adjuvant therapeutics in management of various disease ailments, including obesity, irritable bowel syndrome, lactose intolerance and cancer. To investigate the possible use of *Lactococcus lactis* strains from our collection in treatment of gastrointestinal cancer, we tested them for the ability to arrest proliferation of human colorectal adenocarcinoma cells (Caco-2). Results of the BrdU assay showed that the anti-proliferative activity of *L. lactis* cells is strain-specific. We found that particularly, two strains, *L. lactis* IBB109 and *L. lactis* IBB417, exhibited the most potent inhibitory effect. Moreover, both strains triggered interleukin 18 gene expression, normally inhibited in Caco-2 (cancer) cells. To examine the probiotic potential of the two strains, we tested them for bile salts and acid tolerance, as well as adhesion properties. Both isolates exhibited probiotic potential—they survived in the presence of 0.3% bile salts and tolerated exposure to low pH and osmotic stress. Notably, we found that *L. lactis* IBB417 displayed better adherence to mucus and Caco-2 cells than *L. lactis* IBB109. Additionally, by microdilution tests we confirmed that both strains are sensitive to all nine antibiotics of human and veterinary importance listed by the European Food Safety Authority. Finally, by *in silico* investigations of whole genome sequencing data, we revealed the genetic features of *L. lactis* IBB109 and *L. lactis* IBB417 that can be associated with functional (e.g., adhesion and carbohydrate metabolic genes) and safety (e.g., virulence and antibiotic resistance) aspects of the strains, confirming their health-promoting potential.

## Introduction

Lactic acid bacteria (LAB) are a diverse group of GRAS (generally regarded as safe) Gram-positive microorganisms used in the food industry, especially for production of dairy foods. Certain LAB strains carry probiotic properties and are documented to exert a positive effect on human and animal health. Due to these features, they are considered for use in therapeutic interventions. The majority of probiotics belong to lactobacilli and bifidobacteria, but include also *Lactococcus*, *Streptococcus*, *Pediococcus* strains, among others (Fijan, 2014; Markowiak and Śliżewska, 2017). Some of these bacteria (e.g., *Lacticaseibacillus rhamnosus* GG—basonym *Lactobacillus rhamnosus* GG, *Lacticaseibacillus casei* Shirota—basonyn *Lactobacillus casei*, and *Lactobacillus acidophilus* LA-1) are marketed as probiotic preparations for use as health supplements (Sniffen et al., 2018; Zielińska and Kolożyn-Krajewska, 2018).

Strains with probiotic properties are isolated from various natural sources, such as traditional dairy products (e.g., kefir, oscypek, Iranian Spar), fermented food (e.g., sausages, cabbage, kimchi), raw milk of various origin (e.g., human, cow, goat), and human or animal intestinal microbiota (Zielińska and Kolożyn-Krajewska, 2018). Regardless of the source, the characteristic traits defining probiotic strains include adhesion to intestinal mucosa and/or epithelial layer, resistance to low pH, osmotic stress and bile salts, and the ability to degrade complex carbohydrates (Salminen et al., 2005; Ventura et al., 2012; Markowiak and Śliżewska, 2017).

Among the recognized health-promoting effects of probiotic LAB strains are anti-inflammatory properties, stimulation of the host immune system, competition for nutrients and ecological niches with pathogenic bacteria, inhibition of toxic substance activity, and reduction in lactose intolerance (Saxelin et al., 2005; Markowiak and Śliżewska, 2017). The above-mentioned effects are attained by various pathways/mechanisms and effector molecules. Depending on the site of action, these molecules can be grouped as intracellular factors, such as enzymes (lactase, bile salts hydrolases), metabolites (lactate, short chain fatty acids—SCFAs, bacteriocins), DNA with unmethylated CpG motifs, or surface located determinants (e.g., peptidoglycan, (lipo)teichoic acids, pili, exopolysaccharides, Ser/Thr-rich proteins, S-layer proteins) (Saxelin et al., 2005; Lee et al., 2013; Kim et al., 2017; Wang et al., 2019).

Experimental studies show the beneficial influence of selected LAB strains on the course of certain illnesses related with dysbiosis of the gut microbiota, including irritable bowel syndrome, diarrhoeas, inflammatory bowel disease or vaginal infections (Reid and Bocking, 2003; Markowiak and Śliżewska, 2017). Restoration of microbial balance is often successfully supported by probiotic LAB strains, which produce a plethora of metabolites and bioproducts that maintain gut homeostasis. LAB, provided as dietary supplements, were shown to confer protection against the imbalance of gut microbiota, stimulate the immune defense mechanisms of the host and increase nutrient bioavailability (Zitvogel et al., 2017; Vamanu and Gatea, 2020). Specifically, administration of *Lb. plantarum* DSM 9843 to patients with irritable bowel syndrome reduced amounts of enterococci in fecal samples and provided symptomatic relief (Nobaek et al., 2000). Intake of probiotic strains (*Lb. rhamnosus* GG, *Lb. rhamnosus* Lc705, *Propionibacterium freudenreichii* ssp. *shermanii* JS and *Bifidobacterium animalis* ssp. *lactis* Bb12) in patients with irritable bowel syndrome was found to stabilize intestinal microbiota (Kajander et al., 2008). A plethora of LAB strains are described to induce immune responses in the gut and activate secretion of proinflammatory and regulatory cytokines (e.g., IFN-ƴ, IL-10, IL-12, TNF-α, IL-1β; Matsuzaki and Chin, 2000; Zhong et al., 2014). In a recent study (Hradicka et al., 2020), a mix of lactobacilli strains were shown to increase the level of interleukin 18 in colorectal cancer (CRC) cells, which is recognized as an important cytokine for homeostasis of the gut epithelial cells and prevention of CRC progression (Mager et al., 2016). In turn, LAB strains (*Lb. fermentum* B4655, *Lb. plantarum* B4495, *Lb. casei* B1922, *Lb. bulgaricus* CFR2028 and *Lb. acidophilus* B4496) were described to improve mineral bioavailability during soymilk fermentation, reduce the levels of phytic acid and increase the amount of bioactive isoflavones (Rekha and Vijayalakshmi, 2010).

There is growing evidence on the health-promoting effect of LAB strains in respect to CRC, the third most common malignancy affecting the global human population (Baghbani-Arani et al., 2020; Settanni et al., 2020). Epidemiological studies on cancer patients and populations at increased risk have revealed that consumption of cultured dairy products has an inverse correlation with the risk of CRC (Pala et al., 2011; Zhang et al., 2019). Numerous studies report on the beneficial role of probiotics in treatment of CRC in animals and in preventive or post-operative interventions in humans (Serban, 2014). The anticancer activity of some LAB strains, mainly lactobacilli, is documented also by *in vitro* assays. Cell-free cultures of vaginal isolates, *Lb. acidophilus* 36YL and *Enterococcus faecalis*, displayed anticancer activity against four different human cancer cell lines, including gastric (AGS) and colon (HT-29) cells (Nami et al., 2014a,b). Also, *Lb. paracasei* IMPC2.1 and *Lb. rhamnosus* GG inhibited the proliferation of colon (DLD-1) and gastric (HGC-27) cell lines (Orlando et al., 2012). Notably, data concerning the anti-proliferative effect of *L. lactis* strains are limited. Soluble cell extract of a *L. lactis* ssp. *lactis* strain (L.lac *CF*) was shown to have an antiproliferative effect on the human stomach cancer cell line SNU-1 (Kim et al., 2009). A similar effect was elicited on CRC cells SW480 by the cell wall and cytoplasmic extract of *L. lactis* PTCC 1336 strain (Hosseini et al., 2020).

In light of these studies, LAB strains with confirmed antitumor effects are widely pursued as novel drugs/food supplements for the modulation of gut microbiota in prevention or supplementary treatment of CRC. Yet, application of LAB of unknown genome content may lead, especially in immunocompromised patients, to the development of opportunistic strain or even bacteremia. The intended therapeutic use of LAB strains necessitates confirmation of their biosafety, both at the physiological and genetic level. The evolving era of probiogenomics allows for an in-depth screening of determinants that stand behind the probiotic potential of selected strains, including adhesion to host mucin layer, tolerance to bile salts and acidic conditions, inhibition of pathogens, production of bioactive compounds, ability to metabolize complex carbohydrates.

Our screening for *L. lactis* strains with antiproliferative effects on CRC cells showed that the inhibitory activity is strain-dependent. Particularly, two strains (*L. lactis* IBB109 and *L. lactis* IBB417) exhibited a high inhibitory effect against Caco-2 cells and increased IL-18 production in these cells. We evaluated the resistance of IBB109 and IBB417 to low pH, bile salts and osmolytes, and characterized their adhesive properties as well as sugar catabolic potential. To identify the genetic factors associated with the physiological properties and to determine the biosafety of both strains at sequence level, we performed whole-genome sequencing (WGS). The presence of putative adhesins suggests possible adherence of *L. lactis* IBB109 and IBB417 to mucins, extracellular matrix (ECM) and host epithelial cells. Potential stress resistance pathways and genes that are likely to help them survive in the harsh conditions of the gastrointestinal tract (GIT) were detected. We also identified genes encoding proteins that could stimulate the production of immune cells and whole metabolic pathways that might provide prohealth properties. By confirming the lack of virulence, toxic metabolite and transmissible antibiotic resistance genes, we evaluated the biosafety of both strains. Results of our study support future industrial/therapeutic applications of *L. lactis* IBB109 and *L. lactis* IBB417 strains.

## Materials and Methods

### Bacteria Strains and Growth Conditions

*Lactococcus lactis* strains used in the study derived from the bacterial strain collection of IBB PAS (COLIBB, Poland). All of the tested *L. lactis* strains in this collection were initially isolated from various natural dairy sources (non-commercial products), such as artisanal, home-made products or raw milk and were previously determined to have different RAPD (randomly amplified polymorphic DNA) profiles. Particularly, IBB109 was isolated from raw cow milk and IBB417 from traditionally produced bryndza cheese. All *L. lactis* strains taken for analyses are listed in Table 1. Cells were grown in liquid M17 media (Oxoid) supplemented with 0.5% glucose (GM17) or GM17 solid agar (1.5%) plates at 30°C under aerobic conditions.

**Table 1:** *Lactococcus lactis* strains used in the study.

*L. lactis* strain	Relevant features	Source or references
IBB109	Raw milk isolate	GenBank accession no. CP087600-CP087605
IBB417	Traditional bryndza cheese isolate	GenBank accession no. CP087699-CP087704
IL1403	*L. lactis* subsp. *lactis* wild-type strain	Chopin et al., 1984
IL6288	Prophage-free derivative of *L. lactis* IL1403 strain	Bolotin et al., 2019
TIL448	*L. lactis* subsp. *lactis* NCDO2110, isolated from peas	Le et al., 2013
IBB477	*L. lactis* subsp. *cremoris*, wild-type strain	Radziwiłł-Bieńkowska et al., 2016

### Cell Cultures and Growth Conditions

Human colorectal adenocarcinoma-derived cell lines Caco-2 (ATCC®HTB-37TM) were cultured in conditions recommended by the American Type Culture Collection i.e., at 37°C, 5% CO_2_, 95% humidity in MEM (Minimal Essential Medium; Gibco, Waltham, MA, United States), supplemented with 10% FBS (fetal bovine serum, Sigma-Aldrich, St. Louis, MO, United States), NEAA (1X; non-essential amino acid solution, Sigma-Aldrich, St. Louis, MO, United States), 1 mM sodium pyruvate (Sigma-Aldrich, St. Louis, MO, United States) and addition of penicillin (100 U ml^−1^), and streptomycin (100 μg ml^−1^) (Sigma-Aldrich, St. Louis, MO, United States) in flasks to obtain appropriate density. Cells were then detached by trypsin (Sigma-Aldrich, St. Louis, MO, United States), and their number was counted in the Thoma cell counting chamber. Next, cells were diluted in cell medium to obtain 10^5^ cells ml^−1^. Cells were seeded on 96-well plates at 10^4^ cells per cell and incubated for 24 h. Immediately before application of bacterial cell suspensions, the medium was removed.

### Cultivation of Bacteria for Cell Proliferation Assay

Overnight (o/n) bacterial cultures grown in liquid GM17 were centrifuged (5,500 × *g*, 4°C, 10 min.) and washed twice with phosphate-buffered saline (PBS pH 7.4; BioShop, Burlington, Canada). To determine the concentration of bacterial cell suspensions, serial dilutions were plated on GM17 solid agar and counted after o/n incubation at 30°C. Until that time, the remaining part of bacterial cell suspensions were kept at 4°C o/n. Next, they were centrifuged (5,500 × *g*, 4°C, 10 min.) and resuspended in supplemented MEM without antibiotics to a final concentration of 10^8^ colony forming units (CFUs) ml^−1^. Samples of 100 μl were applied on 96-well plates coated with Caco-2 cells.

### Cell Proliferation Assay

Ninety six-well plates covered with the human colorectal adenocarcinoma-derived cell line Caco-2 were co-incubated with bacterial suspensions (10^7^ CFU per well) for 72 h in optimal conditions for cell culture growth (37°C, 5% CO_2_, 95% humidity). Proliferation of Caco-2 was determined by colorimetric method using the Cell Proliferation Kit, BrdU (Roche) according to manufacturer’s instruction. The assay was performed in 10 technical repeats. Cell cultures incubated without bacteria served as a control.

### Sugar Fermentation Profiles

The sugar catabolic potential was assessed using the API® 50 CHL kit (BioMerieux, France) containing strips with 49 different carbon sources. Strains were prepared and assayed according to the manufacturer’s recommendations. The resulting sugar fermentation pattern was established after 48-h incubation at 37°C in aerobic conditions. Full change in color of the indicator medium was interpreted as a partial sugar utilization. The experiment was performed in triplicate. *L. lactis* IL6288 strain—the prophage-free derivative of the model IL403 strain, served as a control for comparison.

### Adhesion Assays to Polystyrene and Mucin

The ability of the tested bacteria to adhere to abiotic (bare polystyrene) and biotic surfaces (polystyrene plates coated with mucin) was investigated according to Radziwiłł-Bieńkowska et al. (2016). Mucin-covered 96-well plates (Thermo Scientific, Waltham, MA, United States) were prepared by coating each well with type III mucin from porcine stomach (PGM; Sigma-Aldrich, St. Louis, MO, United States) dissolved to a final concentration of 10 mg ml^−1^ in PBS pH 7.4. After the removal of mucin plates were washed twice with PBS (BioShop, Burlington, Canada). Then, 100 μl of bacterial cell suspension at optical density of 1 at 600 nm (OD_600_ 1) was placed in each well of bare or mucin-covered plates and incubated aerobically at 30°C for 3 h. Next, the plates were washed with PBS buffer and stained with 0.22 μm-membrane filtered crystal violet (Scharlau, Sentmenat, Spain). The adhesion was assessed by spectrophotometric measurements of stained cells at OD_583_. Strains with confirmed high adhesion properties to polystyrene (IBB477; Radziwiłł-Bieńkowska et al., 2016) and to mucins (TIL448; Le et al., 2013) were used as positive controls. Each experiment was made in three independent biological replicates with eight technical repetitions.

### Adhesion to Caco-2 Cells

The ability of the tested bacteria to adhere to the Caco-2 cell layer was investigated according to the previously published method (Turpin et al., 2012) with minor modifications. For this purpose, Caco-2 cells were cultured in supplemented MEM with antibiotics (as specified above) on 12-well tissue plates for 14 days at 37°C, 5% CO_2_, 95% humidity. The cell culture MEM medium was changed every 3 days; the last change was for medium without antibiotics. Bacterial cells were grown in GM17 liquid medium for 16 h, then harvested by centrifugation (5,500 × *g*, 4°C, 10 min.), washed twice with cold PBS buffer and resuspended in supplemented MEM without antibiotics. Next, bacterial suspensions were added to each well at a ratio of 10:1 (10 CFU of bacteria per 1 Caco-2 cell). Plates were incubated for 3 h at optimal conditions and then washed three times with PBS to remove unbound bacteria. Caco-2 cells with attached bacteria were gently lysed in PBS with 0.1% TRITON X100 (Sigma-Aldrich, St. Louis, MO, United States). Serial dilutions of recovered bacterial cells (adherent bacteria) were plated on GM17 agar and incubated at 30°C for 48 h for viable count enumeration. The adhesion was expressed as the number of viable adherent bacterial cells (CFU) per 1 Caco-2 cell. Results were compared with those obtained for the control strains—*L. lactis* IL1403 non-adherent strain (negative control) and adherent TIL448 strain. Each experiment was made at least in three independent biological replicates with triplicate technical repetitions.

### Bile Salt, Osmolytes, and Acid Tolerance Assays

Simulated conditions of the gut involving acid stress, bile salts resistance and osmotolerance were examined as previously described (Radziwiłł-Bieńkowska et al., 2017; Aleksandrzak-Piekarczyk et al., 2019) with minor modifications. For bile salt tolerance assay, bacterial suspensions at OD_600_ 0.5 were prepared in 1 ml of PBS pH 7.4 (control), PBS pH 7.4 containing 0.1% (w/v) or 0.3% (w/v) bile salts (Sigma cat. no. B8756) and incubated for 1.5, 3, or 6 h at 37°C. To determine resistance toward acidic stress, bacterial suspensions at OD_600_ 0.5 were prepared in 1 ml of saline solution pH 7.0 (control), 4.0 and 2.5. Next, bacterial cells were centrifuged (6,000 *g*, 5 min, RT) and re-suspended in respective control solutions. Serial dilutions were plated on GM17 solid medium and incubated for 48 h at 30°C. After that time, viable CFUs were determined (per ml) in comparison with control conditions. Osmotic shock characteristics were examined by spot assay. For this o/n, bacterial cultures were plated on GM17 solid plates supplemented with 1.5, 2.0, 2.5, 3.0, 3.5, 4.0, 4.5, 5.0% (w/v) NaCl (Sigma-Aldrich, United States) and incubated at 30°C for 48 h. The results were read by visual inspection of colony growth in comparison with cells plated on GM17 agar lacking NaCl. Each experiment was carried out in three independent biological experiments at two repetitions.

### Antibiotic Susceptibility

The antibiotic susceptibility expressed as a minimal inhibitory concentration (MIC) was determined for *L. lactis* IBB109 and IBB417 using the microdilution method in accordance with the International Organization for Standardization (2010) standard.^1^ The selected antibiotics, namely ampicillin (AM), vancomycin (VA), gentamicin (GM), kanamycin (KM), streptomycin (SM), erythromycin (EM), clindamycin (CM), tetracycline (TC), and chloramphenicol (CL) were recommended for testing of bacterial strains intended for use as feed additives by the European Food Safety Authority (EFSA, 2012).

### Effect of *Lactococcus lactis* IBB109 and IBB417 on Cytokine Gene Expression in Mammalian Cells

Influence of *L. lactis* IBB109 and IBB417 on cytokine (IL-1a, IL-18, and TNFɑ) gene expression levels was evaluated in adenocarcinoma colorectal cell line. For this purpose, 24-h cultures of Caco-2 cell lines were incubated with live bacterial cells from the late stationary growth phase. Both human and bacterial cells were cultured as described earlier in the text. Total RNA was extracted using the Universal RNA/miRNA Purification Kit (EURx, Gdańsk, Poland) as three independent biological replicates with three technical repetitions. The level of expression was defined by RT-qPCR method using specific primer pairs: IL-1aF (5′ cgccaatgactcagaggaaga 3′) and IL-1aR (5′ agggcgtcattcaggatgaa 3′) for IL-1a, IL-18F (5′ atcgcttcctctcgcaacaa 3′) and IL-18R (5′ cttctactggttcagcagccatct 3′) for IL-18 and TNF-F (5′ ctcttctgcctgctgcactttg 3′) and TNF-R (5′ atgggctacaggcttgtcactc 3′) for TNFɑ. Results of the assay were normalized against the reference host gene HPRT using specific primer pair: HPRTF (5′ tatggcgacccgcagccct 3′) and HPRTR (5′ catctcgagcaagacgttcag 3′). Reverse transcription was performed with RevertAid First Strand cDNA Synthesis Kit (Thermo Scientific, Waltham, United States) in conditions recommended by the producer, with oligo d(T)18 primers using 0.9 μg of total RNA for each reaction. The qPCR reaction was performed with the LightCycler 480 system (Roche, Basel, Switzerland) and LightCycler 480 SYBR Green I Master (Roche, Basel, Switzerland) by applying 95°C for 10 min, followed by 45 cycles at 95°C for 15 s, 58°C for 15 s, and 72°C for 15 s. Each reaction was performed in three technical repetitions. After completion of the reaction, its specificity was verified by generating melting curves in a single step at 95°C for 5 s and next from 65°C to 97°C with the plate reading at increments of 0.5°C after a 5-s residence time at each temperature. The relative gene expression was calculated using the 2^-ΔΔCT^ method.

### Isolation and Genomic DNA Sequencing

Total DNA of *L. lactis* IBB109 and IBB417 strain was extracted using a Genomic Maxi AX kit (A&A Biotechnology, Gdańsk, Poland) according to the manufacturer’s recommendations. Isolation was preceded by the incubation of the cell pellet with TES (25 mM TRIS pH 8, 10 mM EDTA, 50 mM saccharose) with lysozyme (8 mg ml^−1^) for 15–30 min. DNA quality control was performed by measuring the absorbance at 260/230 nm, template concentration was determined using Qubit fluorometer (Thermo Fisher Scientific, Waltham, United States), and DNA integrity was analyzed by 0.8% agarose gel electrophoresis and by PFGE using BioRad CHEF-III instrument (BioRad, Hercules, United States). Paired-end sequencing library was constructed using the NEB Ultra II FS Preparation Kit (New England Biolabs, Beverly, United States) according to the manufacturer’s instructions. Library was sequenced using an Illumina MiSeq platform (Illumina, San Diego, United States) with 2 × 300 paired-end reads. Sequence quality metrics were assessed using FASTQC (v0.11.9) (Andrews, 2010) and quality trimmed using fastp (v0.20.0) (Chen et al., 2018). Long reads were obtained using the GridION sequencer (Oxford Nanopore Technologies, Oxford, United Kingdom). Prior to long-read library preparation, genomic DNA was sheared into 30 kb fragments using a 26G needle followed by size selection using Short Read Eliminator kit (Circulomics, United States). Recovered DNA (5 μg) was taken for 1D library construction using SQK-LSK109 kit, and 0.5 μg of final library was loaded into R9.4.1 flowcell and sequenced on the MinION sequencer.

### Genome Assembly

Raw nanopore data were base-called using Guppy v3.2.2 (Oxford Nanopore Technologies, Oxford, UK). After quality filtering using NanoFilt (De Coster et al., 2018) and residual adapter removal using Porechop,^2^ the obtained dataset was quality checked using NanoPlot (De Coster et al., 2018). Long nanopore reads were then assembled in hybrid mode using Unicycler (Wick et al., 2017). The remaining ambiguities in the genome assemblies were verified by PCR amplification of DNA fragments, followed by Sanger sequencing with an ABI3730xl Genetic Analyzer (Life Technologies) using BigDye Terminator Mix v. 3.1 chemistry (Life Technologies). All of the possible sequence errors and mis-assemblies were manually corrected using Seqman software (DNAStar) to obtain the complete nucleotide sequence of bacterial genomes.

### Bioinformatic Analyses

Annotation of coding sequences (CDS) and non-coding RNAs was done using the RAST server (Aziz et al., 2008) and checked by BLAST analysis when needed. Prophage loci in the bacterial genomes were identified using the web-based Phaster tool (Zhou et al., 2011; Arndt et al., 2016). Predicted sequence outputs were classified as intact, questionable or incomplete based on their scores: >90, 70–90, or <70, respectively. Plasmid sequences were compared at the nucleotide level using the Circoletto tool (Darzentas, 2010). KEGG database (Kanehisa et al., 2021) and BlastKOALA automatic annotation server (Kanehisa et al., 2016) were used for functional assignment of the identified genes. Search for bacteriocin-encoding genes was performed using the BAGEL4 web server (de Jong et al., 2006). CRISPRCasFinder was used to search for putative CRISPR arrays within the genome sequences of both strains (Couvin et al., 2018). Subcellular localization of proteins was predicted using PSORTb version 3.0 (Yu et al., 2010). The putative adhesion domains in the encoded amino acid sequences were found based on Pfam database (Mistry et al., 2021) using HmmerWeb version 2.41.1 (Potter et al., 2018). Genome sequences were analyzed for the presence of virulence determinants using the VirulenceFinder 2.0 Server version 2.0.3. The database system was designed to detect homologous sequences for the virulence genes related to *Escherichia coli*, *Listeria*, *Staphylococcus aureus* and *Enterococcus* in WGS data (Malberg Tetzschner et al., 2020). The antibiotic resistance genes (ARGs) were searched using the ResFinder 4.1 Server (Bortolaia et al., 2020), CARD 3.1.3 and RGI 5.2.0 (Jia et al., 2017; Alcock et al., 2020) as well as KEGG database (release 100.0).

### Statistical Analysis

Statistical analyses were performed using GraphPad Prism version 9.2.0 (GraphPad Software, San Diego, CA, United States) using the Student’s *t*-test. A value <0.05 was considered statistically significant. Normal distribution was checked with the Shapiro–Wilk test.

## Results

### Screening of *Lactococcus lactis* Strains for Their Effect on Colorectal Cancer Cell Proliferation

**Figure 1:**
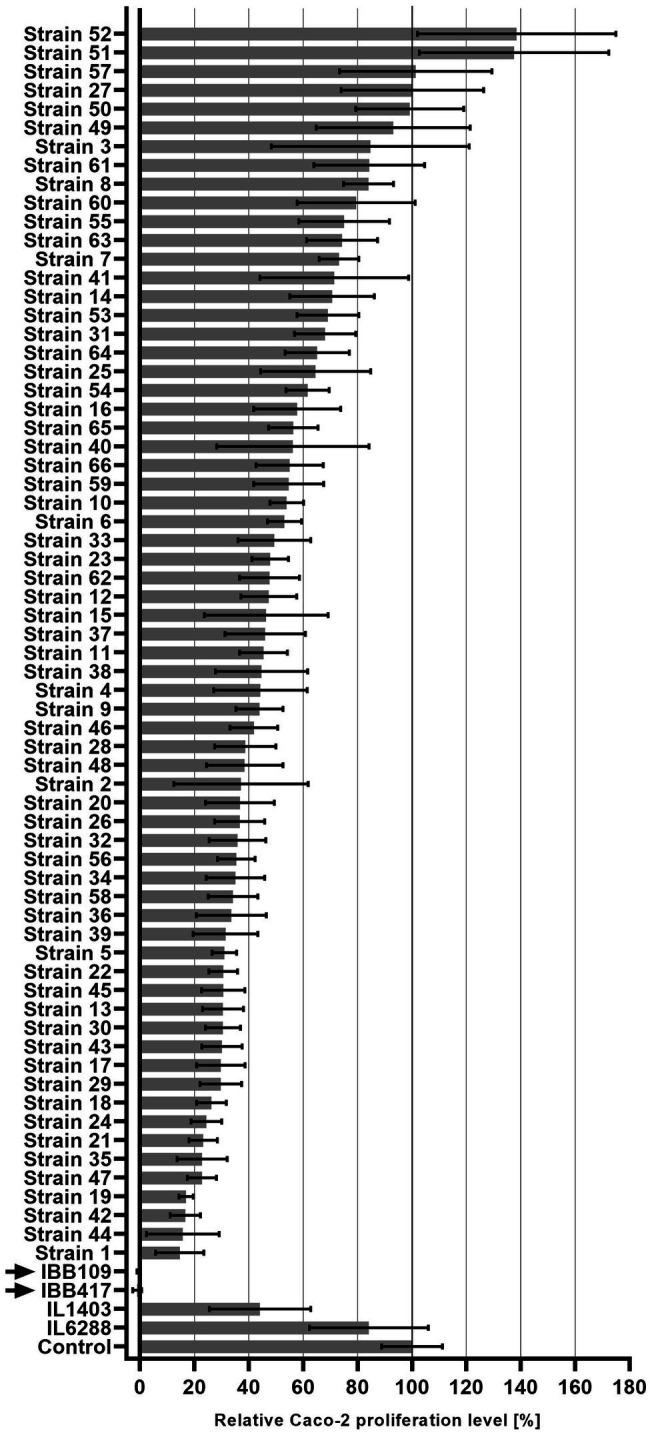
Inhibitory effect of *Lactococcus lactis* strains on Caco-2 cell proliferation. Proliferation of Caco-2 cells incubated with *L. lactis* strains was measured in reference to bacteria-free Caco-2 cell culture (100%) using the BrdU colorimetric assay.

Seventy *Lactococcus* sp. strains were tested for their anti-proliferation activity against the Caco-2 CRC cell line. Proliferation of the cultured cells was shown to be affected in a strain-dependent manner (Figure 1; Supplementary Table S1). Stimulation of Caco-2 cell proliferation was observed for three stains. Seven strains had a weak or no adverse effect on Caco-2 proliferation (80%–100% proliferation level compared to untreated cell culture). Semi-moderate (60%–80% proliferation level) and moderate (40%–60% proliferation level) antiproliferative effect was found for 11 and 19 strains, respectively. Majority of the strains (24 strains) negatively affected Caco-2 cells by inhibition of proliferation to the level of 20%–40%. Finally, strong reduction in Caco-2 cell proliferation to the level of ≤20% was observed for six strains. Among them, two strains—IBB109 and IBB417, displayed the most profound antiproliferative effect. Incubation of Caco-2 cells individually with either IBB109 or IBB417 inhibited almost completely the proliferation of the Caco-2 cell line as detected by the BrdU assay. Based on these observations, IBB109 and IBB417 strains seemed to be of particular interest in potential future anti-cancer prophylactic and therapeutic applications and were selected to be analyzed for their probiotic and biosafety potential, including whole-genome sequencing.

### Bacterial Viability Under Acidic, Bile Salts, and Osmolytic Stress Conditions

Survival and persistence in the harsh conditions of the GIT are important properties defining probiotic bacteria intended for oral ingestion. For the assessment of this probiotic trait, *L. lactis* IBB109 and IBB417 strains were tested for their tolerance against bile salts, low pH and osmotic stress as well as the ability to adhere to abiotic (polystyrene) and biotic (mucin and Caco-2 cells) surfaces.

**Figure 2:**
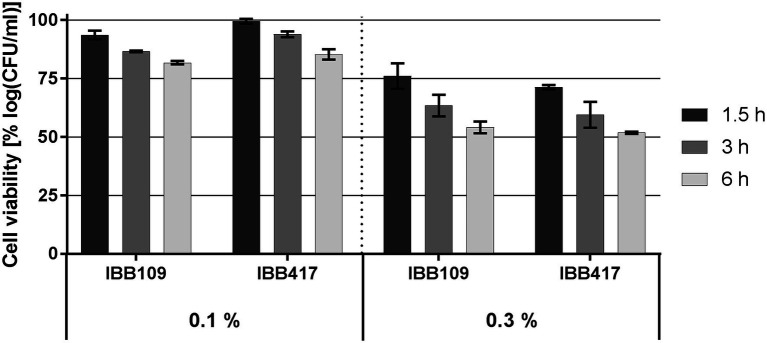
Bile salts tolerance of *L. lactis* strains. Cell survival of *L. lactis* strains was assayed by plate counting after exposure to 0.1% and 0.3% (w/v) bile salts for 1.5, 3, and 6 h. Results were normalized against cell viability under control conditions (without bile salts) and expressed as percent of log CFU ml^−1^.

Survival of IBB109 and IBB417 was inversely correlated with bile salts concentrations and time of exposure (Figure 2; Supplementary Table S2). The best tolerance rate for both strains was noted in 0.1% (w/v) bile salts after a 1.5-h incubation compared to control conditions (incubation without bile salts). Extended exposure to bile stress up to 3 h resulted in the decrease in cell count for both strains and reached at 6 h to an overall relative growth reduction by 18.2% (for IBB109) and 14.7% (for IBB417). Increased bile salts concentration (0.3% w/v) had a more pronounced impact on cell survival. After 6 h, the relative cell viability for both strains declined by approx. 50%.

**Figure 3:**
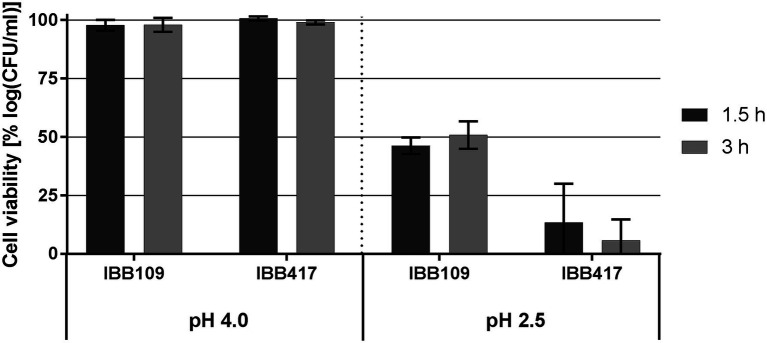
Tolerance to acidic conditions of *L. lactis* strains. Cell survival of *L. lactis* strains was assayed by plate counting after exposure to pH 4 and pH 2.5 for 1.5 and 3 h. Results were normalized against cell viability under control conditions (pH 7) and expressed as percent of log CFU ml^−1^.

At pH 4, the viability of IBB109 and IBB417 was barely affected compared to control conditions (incubation at pH 7; Figure 3; Supplementary Table S3). In turn, exposure to pH 2.5 was highly lethal for IBB417 (13.4% and 5.8% of survival after 1.5 and 3 h). IBB109 tolerated better the same conditions, presenting approx. 50% relative cell survival after 1.5-hour incubation which remained stable at this level over 3 h.

**Figure 4:**
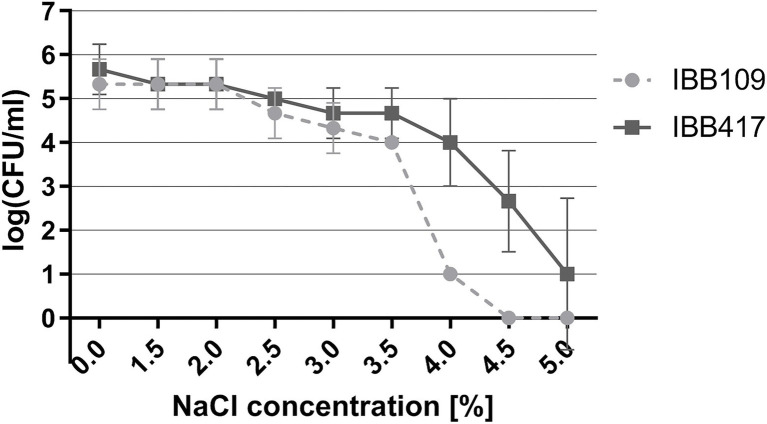
Tolerance of *L. lactis* strains to osmotic stress. Strains were exposed to NaCl (0%–5%; w/v) by spot assay tests. Cell viability is presented as changes in log CFU ml^−1^ values at increasing NaCl concentrations.

Under osmotic stress, the growth of both strains was essentially unaltered in the range of 0.5%–2% NaCl (Figure 4; Supplementary Table S4). The IBB417 strain showed good tolerance to the rising NaCl concentrations and displayed a 1 log decrease in CFU ml^−1^ at 4% NaCl vs. 4.5 log declination of IBB109 viability under the same conditions. Osmotic stress was sustained by IBB417 up to 5% NaCl, albeit with a 4.5 log decline in cell growth. In contrast, IBB109 growth was completely inhibited at 4.5% NaCl. In conclusion, IBB417 was determined as more resistant to conditions of osmotic stress compared to IBB109.

**Figure 5:**
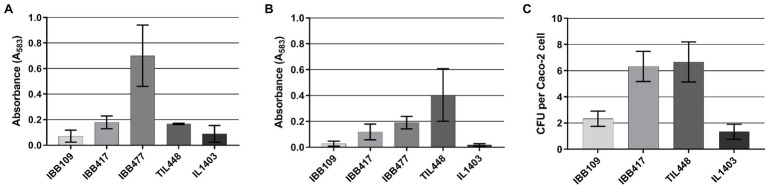
*In vitro* adhesion of *L. lactis* IBB109 and IBB417 strains to **(A)** abiotic and **(B)** biotic surfaces and to **(C)** Caco-2 cells. Adhesion to bare polystyren (abiotic) and mucin-coated polystyrene (biotic) microplates is expressed as optical density (OD_583_ nm) of stained cells. Adhesion to Caco-2 cells is expressed as the number of viable adherent bacterial cells (CFU) per single Caco-2 cell. The mean values ± SD from three independent experiments are shown. Control *L. lactis* strains: IL1403 non-adherent strain (negative control); TIL448 adherent strain to mucin and IBB477 adherent strain to bare polystyrene (positive controls).

In further assays, we determined that IBB109 and IBB417 exhibit different potential to adhere to the tested abiotic (polystyrene) and biotic (mucin) surfaces (Figures 5A,B). The IBB417 strain demonstrated moderate adherence to both polystyrene and mucin as judged by comparison with adherent control strains, i.e., *L. lactis* IBB477 (polystyrene) and TIL448 (mucin). The adhesive properties of IBB109 were weaker than of IBB417, yet still higher than that of the non-adherent control strain (*L. lactis* IL1403).

### Adherence of *Lactococcus lactis* Strains to the Caco-2 Human Colonic Cells

Results of the proliferation study implied direct interaction of *L. lactis* IBB109 and IBB417 with the human colonic cells. To test this hypothesis, both strains were investigated for the ability to adhere to Caco-2 (Figure 5C). Results of this assay showed differences in adherence between IBB109 and IBB417 cells. IBB417 demonstrated good adherence to the differentiated Caco-2 monolayer with an approx. 6:1 ratio (6 CFU to 1 Caco-2 cell) and was comparable to values determined for the highly adhesive TIL448 reference strain. This strongly suggests that IBB417 has good adhesive properties to colonic cells. IBB109 displayed moderate adherence to Caco-2, which was at around 2 CFU per 1 Caco-2 cell (2:1 ratio). Still, both strains could adhere to the Caco-2 cell surface at higher levels than that determined for the non-adherent control strain—IL1403.

### Sugar Catabolic Potential of IBB109 and IBB417

Results of the carbohydrate fermentation profiles are summarized in Supplementary Table S5. Overall, IBB109 and IBB417 presented a similar sugar catabolic spectrum. Each strain was able to fully metabolize 14 out of 49 carbohydrates tested (D-ribose, D-galactose, D-glucose, D-fructose, D-mannose, N-acetylglucosamine, arbutin, esculin ferric citrate, salicin, D-cellobiose, D-maltose, D-lactose, D-saccharose and D-trehalose) and partially utilize starch/amidon (amylolytic activity) and gentiobiose (slight change in medium color). Additionally, a partial fermentation of amygdalin was noted for IBB417. Both strains presented a wider spectrum of sugar metabolism compared to the control model laboratory strain and were in this respect unique in utilizing lactose, saccharose, and arbutin. Neither of the strains was found to ferment D-raffinose or inulin, which are known to have a prebiotic effect. Nonetheless, the obtained results indicate good potential of metabolizing a wide variety of plant-derived sugars or milk sugars that may be encountered in the gut.

### Antibiotic Susceptibility

The inhibitory effect of nine antibiotic substances was tested against using the microdilution method to determine the level of resistance of *L. lactis* IBB109 and IBB417 strains. Based on the obtained results, both strains were determined to have highly similar antibiotic susceptibility profiles (Table 2). The minimal inhibitory values (MICs) against all of the assayed antibiotics were below the break-point values specified by the International Organization for Standardization (2010) standard (see footnote 1) for *L. lactis* strains. As the influence of different antibiotics is species-specific, the obtained data were compared with the antibiotic resistance profile of the ATCC 19435 *L. lactis* control strain lacking antibiotic resistance genes. Both IBB109 and IBB417 exhibited lower MICs than ATCC 19435 and were considered susceptible to all tested antibiotics.

**Table 2:** Antibiotic resistance of *L. lactis* IBB109 and IBB417 strains.

Antibiotic	Minimal inhibitory concentration (MIC)^*^			Break-point values for *L. lactis* strains according to EFSA
	IBB109	IBB417	ATCC 19435 (*L. lactis* Ab^R^-free control strain)	
Gentamicin	1	1–2	0.5–4	32
Kanamycin	8–16	8	2–8	64
Streptomycin	8	8–16	2–16	32
Tetracycline	0.5	0.25–0.5	0.5–2	4
Erythromycin	0.063	0.032–0.063	0.12–0.5	1
Clindamycin	0.032–0.063	0.032	0.25–0.5	1
Chloramphenicol	4	4	4–16	8
Ampicillin	0.25–0.5	0.5	0.5–1	2
Vancomycin	0.25	0.25	0.25–1	4

* MIC values are given in μg ml^−1^.

### *In vitro* Effect of IBB109 and IBB417 on Cytokine Production

**Figure 6:**
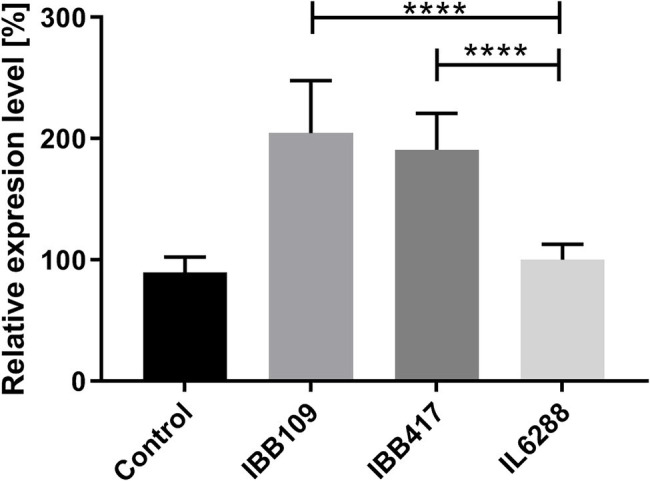
Effect of *L. lactis* IBB109 and IBB417 strains on interleukin 18 gene expression in Caco-2 cells. *L. lactis* IL6288 served as a control strain. Gene expression levels were quantified by real-time PCR (RT-qPCR). Relative gene expression levels were normalized against the HPRT host gene. Error bars represent the SE. For statistical analysis, Student’s *t*-test was used, ^****^p < 0.0001.

Probiotic LAB are known to render various immunomodulatory effects in the host organism (Taverniti and Guglielmetti, 2011; Cristofori et al., 2021). To establish whether *L. lactis* IBB109 and IBB417 can influence host responses, the strains were examined for their ability to induce cytokine production (TNFɑ, IL1a, IL-18) in colorectal adenocarcinoma Caco-2 cells (Supplementary Table S6). Cells treated with IBB109 or IBB417 exhibited significant increase in interleukin-18 gene expression compared to cells without bacteria supplementation (Figure 6). The observed effect was determined to be strain-specific and was not noted for *L. lactis* IL6288 which served as a control. Notably, the gene expression levels for the other tested cytokines (TNFα and IL-1a) were not significantly affected by IBB109 and IBB417 (Supplementary Table S6).

### Analysis of Whole Genome Sequencing Data From IBB109 and IBB417 Strains

*Lactococcus lactis* IBB109 and IBB417 strains, presenting the most prominent activity against human colonic cells, were subjected to whole genome sequencing. Genome sequence analyses of both strains indicated that they belong to the *L. lactis* subsp. *lactis* group. The complete genome of each strain contains a circular double-stranded (ds) DNA chromosome of 2,344,660 bp for IBB109 and 2,380,344 bp for IBB417 with an overall 35.3% GC content typical for this bacterial species (Table 3). The number of chromosomal coding DNA sequences (CDS) determined for IBB109 was 2,388 and 2,424 for IBB 417, where approx. 30% encoded hypothetical products of unknown function in both strains.

**Table 3:** Main features and annotation data for *L. lactis* IBB109 and IBB417 genomes.

Strain	Chromo-some size (bp)	GC content (%)	Number of coding sequences (CDS) in chDNA	Number of CDS in chDNA encoding hypothetical products	tRNA genes	Pro-phage regions	Plasmids
IBB 109	2,344,660	35.3	2,388	690	83	9	pIBB109_AgSa_1 (109 kb) pIBB109_AgSa_2 (55 kb) pIBB109_AgSa_3 (52 kb) pIBB109_AgSa_4 (37 kb) pIBB109_AgSa_5 (35 kb)
IBB 417	2,380,344	35.3	2,424	704	82	10	pIBB417_PrSa_1 (67 kb) pIBB417_PrSa_2 (38 kb) pIBB417_PrSa_3 (9 kb) pIBB417_PrSa_4 (7 kb) pIBB417_PrSa_5 (7 kb)

IBB109 and IBB417 genomes were searched for the presence of prophage sequences (Supplementary Table S7). Our investigation revealed nine prophage-carrying regions for IBB109 (with three intact prophages, six incomplete and one questionable prophage), and ten regions for IBB417 (with two intact, six incomplete and two questionable prophages). The identified intact prophages exhibited the highest sequence similarity to prophages found in the genome of *L. lactis* IL1403 (bIL286, bIL311, bIL312) and *L. lactis* SMQ-86 strain (phage 28201). No confirmed CRISPR/Cas arrays could be detected within the genomes of *L. lactis* IBB417 and IBB109.

Whole genome sequences of both strains were deposited in GenBank under accession no. CP087600-CP087605 (IBB109) and CP087699-CP087704 (IBB417).

### Plasmid Content

IBB109 and IBB417 were found to carry five distinct plasmids each, ranging in size from 7 to 109 kb. All plasmids showed highest similarity to plasmids of other *L. lactis* strains deposited in the GenBank database (Supplementary Figure S1). Each of the plasmids, except for the 109-kb pIBB109_AgSa_1 mega plasmid, carried at least one gene encoding a replication protein having the Rep_3, L_lactis_RepB_C, RepA_N domains. The presence of mega plasmids in *L. lactis* is not a typical feature—out of 140 deposited plasmids in the NCBI database (up to 27 Oct, 2021), only four are larger than 100 kb. Examination of the plasmidic gene content in both strains revealed the presence of genes encoding, among others, transporter proteins, genes involved in metabolic pathways of protein (e.g., casein) degradation or sugar (e.g., lactose) catabolism, exopolysaccharide (EPS) synthesis, resistance to heavy metals, restriction–modification systems, biosynthesis of vitamins and amino acids, and DNA repair. Comparison of plasmid sequences between both strains revealed that two pairs of plasmids, pIBB109_AgSa_2 and pIBB417_PrSa_1 as well as pIBB109_AgSa_4 and pIBB417_PrSa_2, are highly conserved at the nucleotide sequence level (respectively, 99% identity, 100% coverage, and 99% identity, 99% coverage) and in terms of gene arrangement (Supplementary Figure S2). These two pairs of plasmids showed the highest sequence similarity also to other lactococcal plasmids present in databases, whereas pIBB109_AgSa_3 and pIBB417_PrSa_3 seemed to be the most unique.

### Functional Characterization of IBB109 and IBB417 Genomes

Characterization of IBB109 and IBB417 genomes (Supplementary Tables S8, S9) with BlastKOALA revealed that both strains individually encode 199 metabolic pathways. The pathway modules of the two strains (Supplementary Table S10) showed a high degree of similarity. Particularly, no differences in complete pathway modules between the strains were observed. Comparison of complete pathway modules to the IL1403 strain in the KEGG database revealed the lack of five modules present in IBB109 and IBB417. The exclusive metabolic features absent in IL1403 were the complete pathways for glycogen biosynthesis (KEGG entry: M00854), beta-oxidation (Fatty acid metabolism; KEGG entry: M00086), leucine biosynthesis (KEGG entry: M00432), menaquinone (vitamin K2) biosynthesis (KEGG entry: M00116), and the multidrug resistance efflux pump—MepA (KEGG entry: M00705). In regard to vitamins, IBB109 and IBB417, both have complete pathways for biosynthesis of thiamin (vitamin B1), riboflavin (vitamin B2), tetrahydrofolate (vitamin B9), and menaquinone (vitamin K2). Both strains encode enzymes that are dedicated to sugar internalization, through the ATP-binding cassette (ABC) transporters (galactose oligomer/maltooligosaccharide, ribose/autoinducer 2/D-xylose), phosphotransferase systems (sucrose, β-glucoside, mannitol, lactose, cellobiose/diacetylchitobiose), and phosphoenolpyruvate phosphotransferase systems (mannose, fructose). After internalization, sugars can be further utilized by the various carbohydrate metabolic pathways, including at the first step: glycolysis, pentose phosphate pathway and galactose degradation, Leloir pathway.

### Genetic Determinants Encoding Adhesive and Mucoadhesive Properties

The search for extracellular proteins or proteins attached to the cell wall encoded in the *L. lactis* IBB109 genome using PSORTb indicated 57 chromosomal and 11 plasmidic proteins. Similar analysis for IBB417 indicated 60 chromosomally- encoded and four plasmid-encoded proteins of this type. Both genomes have been further searched for the presence of putative domains involved in adhesion to mucus, ECM or to epithelial cells. The following domains have been detected in both strains: MucBP domains [PF06458]; Mub B2-like domain (Mub_B2) [PF17966]; bacterial Ig-like domain—group 3 (Big_3) [PF07523]; collagen binding domain (Collagen-bind) [PF05737]; BspA-type Leucine-rich repeat region (LRR_5) [PF13306]; fibronectin-binding domain (fibronectin-binding protein A N-terminus (FbpA) [PF05833]; Cna protein B-type domain (Cna_B) [PF05738]; bacterial lectin domains (Bact_lectin) [PF18483]; WxL domain surface cell wall-binding (WxL) [PF13731]; clostridial hydrophobic W domain (ChW) [PF07538] and type II secretory pathway pseudopilin (PulG) [PF11773]. The list of all extracellular or attached to cell wall proteins as well as putative adhesins of other localization with domains detected using the Pfam database that are encoded in the genome of IBB109 and IBB417 strain is provided in Supplementary Tables S11, S12, respectively. Based on these analyses, we identified genes encoding putative adhesins which can be selected for further functional studies. Bacterial adhesion to mammalian cells can also be mediated by non-proteinaceous molecules, such as (lipo)teichoic acids. Similar function could possibly be performed by rhamnose-containing cell wall polysaccharides (RhaCWP; Mistou et al., 2016). A set of genes involved in biosynthesis of teichoic acids and rhamnose-containing glycans were found in genomes of both, IBB109 and IBB417.

### Stress Resistance-Related Genes

Probiotics face stress conditions along their transit across the GIT, such as acid, bile, and osmotic stresses. The genome sequences of *L. lactis* IBB109 and IBB417 were screened for genes previously shown to be differentially expressed in cells cultivated under low and optimum pH in *L. lactis* subsp*. cremoris* MG1363 (Carvalho et al., 2013). Upregulated under low pH stress-related genes (such as *dnaK* and *groEL*), H^+^-ATPase subunits, glycolytic genes as well as genes involved in catabolism of amino acids were identified in both analyzed genomes. Additionally, both genomes were searched for genes differentially regulated by bile exposure in *Lacticaseibacillus paracasei* L9 (Ma et al., 2018). The presence of genes of the malolactic enzyme (MLE) pathway as well several other genes upregulated under bile stress—genes involved in various biological processes, including carbon source utilization (mannose/fructose-inducible phosphotransferase system, alpha-glucosidase, and phosphoglycerate mutase), amino acids and peptide metabolism (ABC oligopeptide transport system and biosynthesis of L-lysine), transmembrane transport (multidrug ABC transport system), transcription factors (Xre family), and membrane proteins (phage holin protein, and hemolysin III protein) were confirmed in IBB109 and IBB417 genomes. The *in silico* analysis of genes involved in mechanism for salt tolerance found in genomes of *Lactiplantibacillus plantarum* D31 and T9 strains (Yao et al., 2020) was performed in the tested *L. lactis* strains. Both genomes contained genes involved in mechanisms associated with: (i) the balancing/conservation of intracellular ionic concentrations (Na^+^/H^+^ antiporter and K^+^ transport systems); (ii) absorption, accumulation or synthesis of compatible solutes (glycine betaine ABC transporter OpuAA/OpuAB and choline-betaine ABC transporter BusAA/BusAB, proline synthesis); (iii) transcriptional or response regulators (GntR, Crp/Fnr, LysR families; RNA polymerase sigma factor, S-adenosylmethionine synthetase, DNA-directed RNA polymerase subunit beta, and amino acid permease); and (iv) universal stress response (DnaK, DnaJ, GroES, GroEL). Most of the genes previously found to be involved in acid/bile/osmotic stresses were also identified in the genome of IBB109 and IBB417; however, the resistance to stress must be further explored using transcriptomics analyses to determine the expression rates of the described genes.

### Prohealth-Related Genes

Probiotic bacteria encode a plethora of factors that render health beneficial effects. One of them is an extracellular protein, GroEL, secreted by a range of LAB strains, which was shown to be implicated in adhesion to and immunostimulation of epithelial cells (Bergonzelli et al., 2006; Izquierdo et al., 2009; Gilad et al., 2011). Analysis of the IBB109 and IBB417 genomes revealed that they both encode identical GroEL-like proteins (locus reference: 1_1535829_1534201 for IBB109; locus reference: 1_1528557_1526929 for IBB417), with 100% of identity to the GroEL chaperonin of *L. cremoris* SMQ562 strain. The presence of the GroEL-encoding gene in IBB109 and IBB417 genomes was suspected to contribute to both adherence and increased cytokine gene expression levels in Caco-2 cells.

Antioxidant enzymes, such as superoxide dismutase (SOD), play a significant role in oxidant stress regulation by detoxifying reactive oxygen species. Moreover, SODs produced by LAB strains were shown to decrease gut inflammation and reduce tumor development in mice (Nishikawa et al., 2004; LeBlanc et al., 2011) as well as influence the host immune response by regulating cytokine production (Carroll et al., 2007; LeBlanc et al., 2011). Both of the studied strains were found to encode for manganese superoxide dismutase (MnSOD) genes in their chromosomes (locus reference: 1_1522447_1521827 for IBB109; locus reference: 1_1515175_1514555 for IBB417). Additionally, for IBB417, a second SOD-encoding gene localized on a plasmid (pIBB417_PrSa_3; locus reference: 3_16496_16299) was determined. In both terms, the SOD-encoding genes could be responsible for the observed *in vitro* effects of IBB109 and IBB417 on Caco-2 cells.

Due to the antimicrobial and antagonistic activity, which involves production of various antimicrobial compounds (e.g., organic acids, SCFAs, diacetyl, acetoin, bacteriocins), probiotic bacteria can inhibit the growth of other bacteria, including pathogenic species. Both *L. lactis* strains IBB109 and IBB417 were found to possess complete metabolic pathways to produce lactate, SCFAs (acetate, formate), diacetyl and acetoin (Supplementary Tables S8, S9). Screening for bacteriocins using the BAGEL4 web server revealed that neither strain carried the respective genes.

Anticancer effects have also been associated with production of exopolysaccharides (EPS)–extracellular biopolymers produced by bacteria that form a capsule around the cell or connected loosely as a slime layer (Nwodo et al., 2012; Wu et al., 2021). EPS genes were found only in the IBB109 genome (loci references: 6_15440_14754; 6_16226_15462; 6_16976_16281; Supplementary Table S8).

### Virulence Genes

The safety, in respect to the presence of virulence genes, of IBB109 and IBB417 strains was evaluated *in silico* by comparison of their whole-genome sequences with known virulence genes of *E. coli*, *Enterococcus*, *Listeria*, and *S. aureus*. The virulence factors included *E. coli* Shiga toxin gene and *S. aureus* exoenzyme genes, host immune alteration or evasion genes and toxin genes. Results showed no virulence factors for *L. lactis* IBB109 and IBB417, allowing the conclusion that the genomic sequences of both strains do not include toxic or pathogenic genes related to these four well-known pathogens.

### Antibiotic Resistance Genes

Safety assessment of IBB109 and IBB417 was also evaluated in terms of antibiotic resistance. The results of ARG analysis based on genome sequences data using ResFinder showed that neither of the strains contains antibiotic resistance genes. Similar analysis using CARD and RGI demonstrated that only one gene in IBB417 was homologous to ARG encoding LmrD—a chromosomally encoded efflux pump that confers resistance to lincosamides in *Streptomyces lincolnensis* and *L. lactis* and is responsible for intrinsic resistance (Flórez et al., 2006). The functional analysis performed using KEGG database indicated the presence in both strains of only one, chromosomally encoded, non-transferable ARG—multidrug efflux pump (MepA). These findings, together with results of our antibiotic susceptibility test, indicated that no acquired antibiotic resistance genes were present, and that *L. lactis* IBB109 and IBB417 strains could be recommended as safe.

## Discussion

Probiotic bacteria beneficially influence human health *via* a plethora of mechanisms of action, including modulation of host innate and adaptive immune responses, pathogen exclusion, production of bioactive metabolites or reinforcing gut barrier functions (Guarner et al., 2012).

In the current study, we identified two *L.* strains, IBB109 and IBB417, which exhibit potent antiproliferative activity and stimulate the production of proinflammatory IL-18 cytokine in the colorectal adenocarcinoma cell line (CRC), Caco-2. Results of our work suggest a specific, beneficial effect of both strains in preventing CRC development. By *in silico* mining approach, we screened the genomes of IBB109 and IBB417 strains for genes encoding various proteins and synthesis pathways that have been reported elsewhere to play a role in probiotic function (e.g., molecular chaperones; stress proteins; adhesion proteins; teichoic acids, rhamnose-containing glycans and EPS biosynthesis pathways; glycogen and vitamins biosynthesis pathways, etc.).

Among the important attributes of probiotic strains is their tolerance and viability under the harsh intestinal conditions. Successful passage and persistence of bacterial cells in the GIT is dictated by their ability to withstand low pH (2–4), high bile salts (up to 0.3% w/v) concentrations and high osmolarity (up to 0.3 M; Chowdhury et al., 1996; Prete et al., 2020). In our assays, both IBB109 and IBB417 were fairly resistant to bile salts, low pH and osmotic stress, indicating the potential to survive the conditions of the GIT.

The mechanism used by *L. lactis* species to cope with acid stress is to maintain an optimal intracellular pH by using membrane ATPase FoF1 and the generation of alkaline substances through the catabolism of amino acids (Oliveira et al., 2017). Another acid stress response mechanism is related to proteins that play a key role in general stress response, such as small heat shock proteins, chaperonins, and universal stress proteins. All of these genes have been identified in both analyzed genomes.

The process by which probiotic bacteria could survive bile stress is complex and still unclear. Based on previous investigations, the ability of probiotic strains to survive in the presence of bile salts is linked with production specific enzymes, bile salts hydrolyses (BSH), which deconjugate the bile acids making them accessible for other cellular processes and reducing their toxic effect (Floch et al., 1972; Gilliland and Speck, 1977; Tannock et al., 1989). Genes encoding BSH proteins were not found in the IBB109 and IBB417 genomes, suggesting that the observed tolerance is related to other factors, possibly the presence of the complete glycogen biosynthesis pathway or the malolactic enzyme (MLE) pathway which was shown to be the primary bile tolerance mechanism in *L. paracasei* L9, enhancing resistance through cytoplasm alkalinization (Ma et al., 2018). Among LAB, complete glycogen metabolic pathways are present only in selected species predominantly associated with mammalian hosts or natural environments. *In vitro* and *in vivo* studies established the significance of glycogen biosynthesis not only on bile tolerance but also on growth and sugar utilization as well as the competitive retention of *L. acidophilus* in the mouse intestinal tract, demonstrating that the ability to synthesize intracellular glycogen contributes to gut fitness and retention among probiotic microorganisms (Goh and Klaenhammer, 2014).

Bacterial cells encounter osmotic stress in various settings, including the natural environment, industrial locations or the upper part of the small intestine (Le Marrec, 2011). Both IBB109 and IBB417 displayed relatively good viability in the presence of NaCl (up to 3.5%) and were found to carry putative genes that based on the literature data (Yao et al., 2020) might contribute to the strain-specific response to salt stress by making an osmotic upshift, maintaining the cytosolic redox status and regulating intracellular metabolism. Among the factors conditioning osmotic tolerance, are osmoprotectants, small organic compounds that protect cells from increased membrane tension. Their accumulation in the cytoplasm stimulates the growth of LAB strains in hyperosmotic conditions (Piuri et al., 2003; Le Marrec et al., 2007). It has been suggested that the ability to resist osmotic stress at least for LAB is strain-specific and related rather to the uptake of osmoprotectants from the environment than to synthesis by intrinsic enzymatic machinery (Robert et al., 2000). The recognition and uptake of osmolytes are largely influenced by the presence and the efficiency of specific transporter systems. Both IBB109 and IBB417 were found to possess two putative osmoprotectant transport systems annotated as glycine betaine ABC transporter OpuAA/OpuAB and choline-betaine ABC transporter BusAA/BusAB. Glycine betaine (GB) is commonly present in food products and plant-derived material, such as meat, bovine milk whey, sugar beets and is one of the most widely accumulated organic osmolytes in nature that stimulates bacterial growth. Growth improvement under osmotic stress was also reported for certain LAB strains in the presence of choline (Kets et al., 1994; Robert et al., 2000; Baliarda et al., 2003). The compound itself is not an osmoprotectant, but can be converted to betaine through the choline-glycine betaine pathway identified in some LAB (Robert et al., 2000). The activity of OpuA/BusA systems in LAB activity was shown to be induced in highly osmotic conditions and involve uptake of glycine betaine leading to growth stimulation (Bouvier et al., 2000; Obis et al., 2001). According to previous studies, the presence of extracellular choline does not influence the growth of *L. lactis* strains under hyperosmotic stress (Obis et al., 1999; Uguen et al., 1999), suggesting that in this species choline is not converted to betaine. Therefore, additional experimental studies are needed to examine the functionality and substrate specificity of the identified osmoprotectant uptake system(s) (particularly choline-betaine ABC transporter BusAA/BusAB) in IBB109 and IBB417.

Adherence to epithelial cells is a highly sought feature of bacterial strains with probiotic potential which can serve a protective role against enteropathogens by competition for host cell binding sites (Oliveira et al., 2017). The ability to attach to the host intestinal epithelial layer or mucus may prolong transient colonization and consequently, increase the fitness of probiotic strains in exerting the pro-health effects in the gut. Mucus is among the main components of the gut barrier that serves in protection of the host against e.g., chemical compounds and pathogens. Bacterial cells that adhere to polystyrene usually adhere well to biotic surfaces, including mucus (Aleksandrzak-Piekarczyk et al., 2016). In our assays, *L. lactis* IBB109 and IBB417 presented from low (IBB109) to moderate (IBB417) adhesion to both mucus and polystyrene, but bound with a fairly high (IBB417) or moderate (IBB109) efficiency to Caco-2 cells. Adhesion of bacterial cells to intestinal surfaces can be mediated by several factors, including proteinases (Radziwiłł-Bieńkowska et al., 2017), mucus-binding proteins (Boekhorst et al., 2006), fibronectin- and collagen-binding proteins (Muscariello et al., 2020) or pili (Meyrand et al., 2013). Our probiogenomics analyses revealed the presence of genes encoding extracellular or attached to cell wall proteins as well as putative adhesins of other localization. These proteins contain domains involved in adhesion to mucus, but also to ECM or to epithelial cells. Results of adhesion assay to Caco-2 cells indicated that most probably adhesion to intestinal epithelial cells does not occur *via* mucus-binding proteins but is related to other protein domains (or non-proteinaceous factors) detected for *L. lactis* IBB109 and IBB417 strains.

A genetic factor that has not been detected in our search for determinants encoding adhesive and mucoadhesive properties, but is encoded in the genomes of both strains, is chaperonin GroEL, an intracellular/surface moonlighting protein, which, apart from its role in protein folding, is believed to be involved in bacterial adhesion to mucin and epithelial cells (Bergonzelli et al., 2006; Lippolis et al., 2013 and references within). Such proteins do not contain signal sequences for secretion or known sequence motifs for binding to the cell surface, so in most cases it is not known how these proteins are secreted or how they become attached to the cell surface (Jeffery, 2018). The presence of the GroEL-encoding gene in the genomes of IBB109 and IBB417 could account for adhesive properties that we observed for both strains.

Numerous studies report that wall teichoic acids (WTA) associated with the cell surface of LAB can also play a role in adhesion to intestinal epithelial cells (Op den Camp et al., 1985; Granato et al., 1999). Similar function to WTA is assigned to rhamnose-containing cell wall polysaccharides (RhaCWP; Mistou et al., 2016). Rhamnose-containing glycans were shown to be involved in processes that affect host-pathogen interactions, including adhesion and biofilm formation in fungi (Martinez et al., 2012). Homologues of biosynthesis genes of wall teichoic acids (WTA) and rhamnose-containing glycans were found in both of the analyzed lactococcal genomes.

We have shown that IBB109 and IBB417 exhibit a strong anti-proliferative and significant immunomodulatory effect on colorectal adenocarcinoma Caco-2 cells *in vitro*. The level of cancer cell inhibition can be due to different factors, e.g., adhesion, SCFAs or exopolysaccharide production (Thirabunyanon and Hongwittayakorn, 2013; Mantzourani et al., 2019; Wu et al., 2021). It has been argued that prolonged duration of LAB strains in the gut due to adherence to intestinal cells promotes the action of their secondary metabolites, including SCFAs. SCFAs were found to counteract the deleterious activity of histone deacetylase (HDCA) implicated in cancer development and progression (Glozak et al., 2005; Zhong et al., 2014). A study by Thirabunyanon and Hongwittayakorn (2013) has shown that LAB strains exhibiting adhesive ability coupled with SCFAs production inhibited Caco-2 cell proliferation. Waldecker et al. (2008) demonstrated that inhibition of colon cell lines is specifically related to butyrate. IBB417 and IBB109 displayed, respectively, strong and moderate adhesion to Caco-2 cells and were determined to encode for the complete pathways for lactate and SCFAs (acetate and formate) synthesis. Both of these features could account for the strong inhibitory effect on CRC cells *in vitro*. Lactate is a powerful antimicrobial factor that inhibits the growth of pathogens and participates in the trophic chain between microbial communities, because it favors the growth of bacteria that consume lactate and subsequently generate SCFAs (Louis and Flint, 2017). Despite the fact that the complete set of well-characterized key synthesis genes necessary for butyrate production was not identified in the IBB109 and IBB417 genomes, the possibility of butyrate synthesis cannot be excluded *via* alternative pathways. It is postulated that unknown genes may contribute to the formation of SCFAs (Zhao et al., 2019) as was also in the case of the butyrogenic capability of *L. plantarum* which is highly dependent on the substrate type and for a long time has not been assigned to any specific metabolic pathway (Botta et al., 2017).

Exposition of bacterial molecular structures (cell-anchored or secreted) known as MAMPs (microbially associated molecular patterns) to epithelial cell receptors induces signaling pathways that lead to immune stimulation (Lebeer et al., 2010). Despite the lack of a direct link between adhesion and immune responses, studies show that bacteria which adhere to intestinal surfaces can affect the host immune system, particularly through the gut-associated lymphoid tissue (GALT). Close contact with the gut epithelial cells allows components anchored in the cell wall or secreted by the probiotic bacteria to induce signaling pathways that stimulate the immune cells. Our studies indicate that *L. lactis* IBB109 and IBB417 strains may trigger an immune response in CRC cell lines as they were found to significantly increase the level of proinflammatory IL-18 gene expression. Intestinal epithelial cells (IEC) naturally produce IL-18 at a relatively constant level, which is considered a crucial factor in inhibiting CRC tumor development, tumor reduction and maintaining mucosal equilibrium (Mager et al., 2016; Hradicka et al., 2020). In CRC cells, the IL-18 level is significantly reduced or not detectable (Feng et al., 2020). Such phenomenon is postulated to be a form of escape of cancer cells from the host’s immune system. We suggest that the elevated levels of IL-18 gene expression upon treatment of Caco-2 cells with IBB109 and IBB417 may contribute to the stimulation of the immune system, leading to the recognition of cancer cells by the host organism, and thus, early prevention of disease development.

The ability to antagonize or competitively exclude pathogens is a crucial probiotic trait achieved by bacterial cells upon secretion of antimicrobial substances. LAB produce different antimicrobial components, such as organic acids, hydrogen peroxide, carbon peroxide, diacetyl, low molecular weight antimicrobial substances, bacteriocins and adhesion inhibitors. The presence of genes and pathways dedicated to some of these compounds, such as organic acids (lactate, acetate, formate), diacetyl, acetoin, were detected in the analyzed lactococcal genomes.

The fitness of bacteria to particular niches, such as the GIT, is favored by the ability to utilize various carbon sources. In our assays, IBB109 and IBB417 presented essentially the same carbohydrate fermentation pattern. Of particular importance is the ability of both strains to degrade sugars found in ingested foods, such as milk (lactose), cereals (e.g., maltose, esculin), or plant material (e.g., fructose, arbutin, sucrose) (Gänzle and Follador, 2012), which may support their survival in the intestine.

Additional beneficial, prohealth effects may derive from the presence of genes encoding complete synthesis pathways for essential vitamins of the B and K groups. Most B group vitamins are directly involved in energy metabolism (LeBlanc et al., 2017) and play important roles in the maintenance of immune homeostasis (Yoshii et al., 2019). When produced in the GIT, vitamins may be utilized by the host or by intestinal bacteria which are unable to synthesize them. Vitamins are normally stored inside the cells and released by direct diffusion *via* specific transporters in the cell membrane or cellular lysis, e.g., inside the GIT of the host. Thus, vitamin producing strains are good candidates for *in situ* delivery of vitamins to consumers.

In summary, we showed that two *L. lactis* strains, IBB109 and IBB417, exhibit potent a proliferation inhibition and increased interleukin 18 (IL-18) expression in human colon cancer cells (Caco-2). Physiological assays revealed the health-promoting, metabolic and biosafety features of *L. lactis* IBB109 and IBB417. By whole-genome sequencing and *in silico* analyses, we evaluated the presence of genes and pathways that may be responsible for their *in vitro* effects. Our studies underlie the multi-method approach that can be adapted when examining the potential of bacterial strains in developing innovative therapeutic microbial-based products.

## Data Availability Statement

The original contributions presented in the study are included in the article and Supplementary Materials file; further inquiries can be directed to the corresponding author.

## Author Contributions

PS performed all experimental works, statistical and part of bioinformatic analyses, prepared figures and tables, and wrote parts of the manuscript. MK performed bioinformatics analyses, prepared figures and tables, and wrote sections of the manuscript. JB co-supervised the work and provided conceptual insights. AS was responsible for the experimental concept, critically reviewed and analyzed all data, prepared figures and tables, and wrote the main text of the manuscript. All authors contributed to the article and approved the submitted version.

## Funding

This research was financed by the statutory funds of the Institute of Biochemistry and Biophysics, Polish Academy of Sciences.

## Conflict of Interest

The authors declare that the research was conducted in the absence of any commercial or financial relationships that could be construed as a potential conflict of interest.

## Publisher’s Note

All claims expressed in this article are solely those of the authors and do not necessarily represent those of their affiliated organizations, or those of the publisher, the editors and the reviewers. Any product that may be evaluated in this article, or claim that may be made by its manufacturer, is not guaranteed or endorsed by the publisher.

## Footnotes

^1^ https://www.iso.org/standard/46434.html

^2^ https://github.com/rrwick/Porechop

## References

[ref1] Alcock B. P. Raphenya A. R. Lau T. T. Y. (2020). CARD 2020: antibiotic resistome surveillance with the comprehensive antibiotic resistance database. Narrative 48, D517–D525. doi: 10.1093/nar/gkz935, PMID: 31665441 PMC7145624

[ref2] Aleksandrzak-Piekarczyk T. Koryszewska-Bagińska A. Grynberg M. Nowak A. Cukrowska B. Kozakova H.. (2016). Genomic and functional characterization of the unusual pLOCK0919 plasmid harboring the spaCBA pili cluster in *Lactobacillus casei* LOCK 0919. Genome Biol. Evol. 8, 202–217. doi: 10.1093/gbe/evv247, PMID: 26637469 PMC4758243

[ref3] Aleksandrzak-Piekarczyk T. Puzia W. Żylińska J. Cieśla J. Gulewicz K. A. Bardowski J. K.. (2019). Potential of *Lactobacillus plantarum* IBB3036 and *Lactobacillus salivarius* IBB3154 to persist in chicken after *in ovo* delivery. Microbiology 8:e00620. doi: 10.1002/mbo3.620, PMID: 29575743 PMC6341040

[ref4] Andrews S. (2010). FastQC: A Quality Control Tool for High Throughput Sequence Data [Online]. Available at: http://www.bioinformatics.babraham.ac.uk/projects/fastqc/

[ref5] Arndt D. Grant J. Marcu A. Sajed T. Pon A. Liang Y.. (2016). PHASTER: a better, faster version of the PHAST phage search tool. Narrative 44, W16–W21. doi: 10.1093/nar/gkw387, PMID: 27141966 PMC4987931

[ref6] Aziz R. K. Bartels D. Best A. A. DeJongh M. Disz T. Edwards R. A.. (2008). The RAST server: rapid annotations using subsystems technology. BMC Genomics 9:75. doi: 10.1186/1471-2164-9-75, PMID: 18261238 PMC2265698

[ref7] Baghbani-Arani F. Asgary V. Hashemi A. (2020). Cell-free extracts of *Lactobacillus acidophilus* and *Lactobacillus delbrueckii* display antiproliferative and antioxidant activities against HT-29 cell line. Nutr. Cancer 72, 1390–1399. doi: 10.1080/01635581.2019.1685674, PMID: 31707847

[ref8] Baliarda A. Robert H. Jebbar M. Blanco C. Deschamps A. Le Marrec C. (2003). Potential osmoprotectants for the lactic acid bacteria *Pediococcus pentosaceus* and *Tetragenococcus halophila*. Int. J. Food Microbiol. 84, 13–20. doi: 10.1016/s0168-1605(02)00388-4, PMID: 12781949

[ref9] Bergonzelli G. E. Granato D. Pridmore R. D. Marvin-Guy L. F. Donnicola D. Corthesy-Theulaz I. E. (2006). GroEL of *Lactobacillus johnsonii* La1 (NCC 533) is cell surface associated: potential role in interactions with the host and the gastric pathogen, *Helicobacter pylori*. Infect. Immun. 74, 425–434. doi: 10.1128/IAI.74.1.425-434.2006, PMID: 16368998 PMC1346591

[ref10] Boekhorst J. Helmer Q. Kleerebezem M. Siezen R. J. (2006). Comparative analysis of proteins with a mucus-binding domain found exclusively in lactic acid bacteria. Microbiology 152, 273–280. doi: 10.1099/mic.0.28415-0, PMID: 16385136

[ref11] Bolotin A. Aucouturier A. Sorokin A. Bidnenko E. (2019). Genomic sequence of the prophage-free *Lactococcus lactis* strain IL6288. Microbiol. Resour. Announc. 8, e01545–e01618. doi: 10.1128/MRA.01545-18, PMID: 30637406 PMC6318377

[ref12] Bortolaia V. Kaas R. F. Ruppe E. Roberts M. C. Schwarz S. Cattoir V.. (2020). ResFinder 4.0 for predictions of phenotypes from genotypes. J. Antimicrob. Chemother. 75, 3491–3500. doi: 10.1093/jac/dkaa345, PMID: 32780112 PMC7662176

[ref13] Botta C. Acquadro A. Greppi A. Barchi L. Bertolino M. Cocolin L.. (2017). Genomic assessment in *Lactobacillus plantarum* links the butyrogenic pathway with glutamine metabolism. Sci. Rep. 7:15975. doi: 10.1038/s41598-017-16186-8, PMID: 29162929 PMC5698307

[ref14] Bouvier J. Bordes P. Romeo Y. Fourçans A. Bouvier I. Gutierrez C. (2000). Characterization of OpuA, a glycine-betaine uptake system of *Lactococcus lactis*. J. Mol. Microbiol. Biotechnol. 2, 199–205. 10939245

[ref15] Carroll I. M. Andrus J. M. Bruno-Barcena J. M. Klaenhammer T. R. Hassan H. M. Threadgill D. S. (2007). Anti-inflammatory properties of *Lactobacillus gasseri* expressing manganese superoxide dismutase using the interleukin 10-deficient mouse model of colitis. Am. J. Physiol. Gastrointest. Liver Physiol. 293, G729–G738. doi: 10.1152/ajpgi.00132.2007, PMID: 17640978

[ref16] Carvalho A. L. Turner D. L. Fonseca L. L. Solopova A. Catarino T. Kuipers O. P.. (2013). Metabolic and transcriptional analysis of acid stress in *Lactococcus lactis*, with a focus on the kinetics of lactic acid pools. PLoS One 8:e68470. doi: 10.1371/journal.pone.0068470, PMID: 23844205 PMC3700934

[ref17] Chen S. Zhou Y. Chen Y. Gu J. (2018). Fastp: an ultra-fast all-in-one FASTQ preprocessor. Bioinformatics 34, i884–i890. doi: 10.1093/bioinformatics/bty560, PMID: 30423086 PMC6129281

[ref18] Chopin A. Chopin M.-C. Moillo-Batt A. Langella P. (1984). Two plasmid-determined restriction and modification systems in *Streptococcus lactis*. Plasmid 11, 260–263. doi: 10.1016/0147-619X(84)90033-7, PMID: 6087394

[ref19] Chowdhury R. Sahu G. K. Das J. (1996). Stress response in pathogenic bacteria. J. Biosci. 21, 149–160. doi: 10.1007/BF02703105

[ref20] Couvin D. Bernheim A. Toffano-Nioche C. Touchon M. Michalik J. Néron B.. (2018). CRISPRCasFinder, an update of CRISRFinder, includes a portable version, enhanced performance and integrated search for Cas proteins. Narrative 46, W246–W251. doi: 10.1093/nar/gky425, PMID: 29790974 PMC6030898

[ref21] Cristofori F. Dargenio V. N. Dargenio C. Miniello V. L. Barone M. Francavilla R. (2021). Anti-inflammatory and immunomodulatory effects of probiotics in gut inflammation: a door to the body. Front. Immunol. 12:578386. doi: 10.3389/fimmu.2021.578386, PMID: 33717063 PMC7953067

[ref22] Darzentas N. (2010). Circoletto: visualizing sequence similarity with Circos. Bioinformatics 26, 2620–2621. doi: 10.1093/bioinformatics/btq484, PMID: 20736339

[ref23] De Coster W. D’Hert S. Schultz D. T. Cruts M. Van Broeckhoven C. (2018). NanoPack: visualizing and processing long-read sequencing data. Bioinformatics 34, 2666–2669. doi: 10.1093/bioinformatics/bty149, PMID: 29547981 PMC6061794

[ref24] de Jong A. van Hijum S. A. Bijlsma J. J. Kok J. Kuipers O. P. (2006). BAGEL: a web-based bacteriocin genome mining tool. Narrative 34, W273–W279. doi: 10.1093/nar/gkl237, PMID: 16845009 PMC1538908

[ref25] EFSA (2012). Guidance on the assessment of bacterial susceptibility to antimicrobials of human and veterinary importance. EFSA J. 10:2740. doi: 10.2903/j.efsa.2012.2740

[ref26] Feng X. Zhang Z. Sun P. Song G. Wang L. Sun Z.. (2020). Interleukin-18 is a prognostic marker and plays a tumor suppressive role in colon cancer. Dis. Markers 2020:6439614. doi: 10.1155/2020/6439614, PMID: 33294056 PMC7714607

[ref27] Fijan S. (2014). Microorganisms with claimed probiotic properties: an overview of recent literature. Int. J. Environ. Res. Public Health 11, 4745–4767. doi: 10.3390/ijerph110504745, PMID: 24859749 PMC4053917

[ref28] Floch M. H. Binder H. J. Filburn B. Gershengoren W. (1972). The effect of bile acids on intestinal microflora. Am. J. Clin. Nutr. 25, 1418–1426. doi: 10.1093/ajcn/25.12.1418 4344803

[ref29] Flórez A. B. de Los Reyes-Gavilán C. G. Wind A. Mayo B. Margolles A. (2006). Ubiquity and diversity of multidrug resistance genes in *Lactococcus lactis* strains isolated between 1936 and 1995. FEMS Microbiol. Lett. 263, 21–25. doi: 10.1111/j.1574-6968.2006.00371.x 16958846

[ref30] Gänzle M. G. Follador R. (2012). Metabolism of oligosaccharides and starch in lactobacilli: a review. Front. Microbiol. 3:340. doi: 10.3389/fmicb.2012.00340, PMID: 23055996 PMC3458588

[ref31] Gilad O. Svensson B. Viborg A. H. Stuer-Lauridsen B. Jacobsen S. (2011). The extracellular proteome of *Bifidobacterium animalis* subsp. *lactis* BB-12 reveals proteins with putative roles in probiotic effects. Proteomics 11, 2503–2514. doi: 10.1002/pmic.201000716, PMID: 21598393

[ref32] Gilliland S. E. Speck M. L. (1977). Deconjugation of bile acids by intestinal lactobacilli. Int. J. Bacteriol. 33, 15–18. doi: 10.1128/aem.33.1.15-18.1977 PMC170567 13710

[ref33] Glozak M. A. Sengupta N. Zhang X. Seto E. (2005). Acetylation and deacetylation of non-histone proteins. Gene 363, 15–23. doi: 10.1016/j.gene.2005.09.010 16289629

[ref34] Goh Y. J. Klaenhammer T. R. (2014). Insights into glycogen metabolism in *Lactobacillus acidophilus*: impact on carbohydrate metabolism, stress tolerance and gut retention. Microb. Cell Factories 13:94. doi: 10.1186/s12934-014-0094-3, PMID: 25410006 PMC4243779

[ref35] Granato D. Perotti F. Masserey I. Rouvet M. Golliard M. Servin A.. (1999). Cell surface-associated lipoteichoic acid acts as an adhesion factor for attachment of *Lactobacillus johnsonii* La1 to human enterocyte-like Caco-2 cells. Appl. Environ. Microbiol. 65, 1071–1077. doi: 10.1128/AEM.65.3.1071-1077.1999, PMID: 10049865 PMC91146

[ref36] Guarner F. Khan A. G. Garisch J. Eliakim R. Gangl A. Thomson A.. (2012). World gastroenterology organisation global guidelines: probiotics and prebiotics. J. Clin. Gastroenterol. 46, 468–481. doi: 10.1097/MCG.0b013e3182549092 22688142

[ref37] Hosseini S. S. Goudarzi H. Ghalavand Z. Hajikhani B. Rafeieiatani Z. Hakemi-Vala M. (2020). Anti-proliferative effects of cell wall, cytoplasmic extract of *Lactococcus lactis* and nisin through down-regulation of cyclin D1 on SW480 colorectal cancer cell line. Iran. J. Microbiol. 12, 424–430. doi: 10.18502/ijm.v12i5.4603, PMID: 33603997 PMC7867695

[ref38] Hradicka P. Beal J. Kassayova M. Foey A. Demeckova V. (2020). A novel lactic acid bacteria mixture: macrophage-targeted prophylactic intervention in colorectal cancer management. Microorganisms 8:387. doi: 10.3390/microorganisms8030387, PMID: 32168834 PMC7142725

[ref39] International Organization for Standardization (2010). ISO 10932 [IDF 223:2010]. Milk and milk products—determination ofthe minimal inhibitory concentration (MIC) ofantibiotics applicable to bifidobacteria and non-enterococcal lactic acid bacteria (LAB). International Organization for Standardization, Geneva, Switzerland. Available at: https://www.iso.org/standard/46434.html

[ref40] Izquierdo E. Horvatovich P. Marchioni E. Aoude-Werner D. Sanz Y. Ennahar S. (2009). 2-DE and MS analysis of key proteins in the adhesion of *Lactobacillus plantarum*, a first step toward early selection of probiotics based on bacterial biomarkers. Electrophoresis 30, 949–956. doi: 10.1002/elps.200800399, PMID: 19309013

[ref41] Jeffery C. (2018). Intracellular proteins moonlighting as bacterial adhesion factors. AIMS Microbiol. 4, 362–376. doi: 10.3934/microbiol.2018.2.362, PMID: 31294221 PMC6604927

[ref42] Jia B. Raphenya A. R. Alcock B. Waglechner N. Guo P. Tsang K. K.. (2017). CARD 2017: expansion and model-centric curation of the comprehensive antibiotic resistance database. Narrative 45, D566–D573. doi: 10.1093/nar/gkw1004, PMID: 27789705 PMC5210516

[ref43] Kajander K. Myllyluoma E. Rajilić-Stojanović M. Kyrönpalo S. Rasmussen M. Järvenpää S.. (2008). Clinical trial: multispecies probiotic supplementation alleviates the symptoms of irritable bowel syndrome and stabilizes intestinal microbiota. Aliment. Pharmacol. Ther. 27, 48–57. doi: 10.1111/j.1365-2036.2007.03542.x, PMID: 17919270

[ref44] Kanehisa M. Sato Y. Kawashima M. (2021). KEGG mapping tools for uncovering hidden features in biological data. Protein Sci. 31, 47–53. doi: 10.1002/pro.4172, PMID: 34423492 PMC8740838

[ref45] Kanehisa M. Sato Y. Morishima K. (2016). BlastKOALA and GhostKOALA: KEGG tools for functional characterization of genome and metagenome sequences. J. Mol. Biol. 428, 726–731. doi: 10.1016/j.jmb.2015.11.006, PMID: 26585406

[ref46] Kets E. P. W. Galinski E. A. de Bont J. A. M. (1994). Carnitine: a novel compatible solute in *Lactobacillus plantarum*. Arch. Microbiol. 162, 243–248. doi: 10.1007/BF00301845

[ref47] Kim K. W. Kang S. S. Woo S. J. Park O.-J. Ahn K. B. Song K.-D.. (2017). Lipoteichoic acid of probiotic *Lactobacillus plantarum* attenuates poly I:C-induced IL-8 production in porcine intestinal epithelial cells. Front. Microbiol. 8:1827. doi: 10.3389/fmicb.2017.01827, PMID: 28983294 PMC5613100

[ref48] Kim S. Y. Kim J. E. Lee K. W. Lee H. J. (2009). *Lactococcus lactis* ssp. *lactis* inhibits the proliferation of SNU-1 human stomach cancer cells through induction of G0/G1 cell cycle arrest and apoptosis via p53 and p21 expression. Ann. N. Y. Acad. Sci. 1171, 270–275. doi: 10.1111/j.1749-6632.2009.04721.x, PMID: 19723065

[ref500] Lebeer S. Vanderleyden J. De Keersmaecker S. C. (2010). Host interactions of probiotic bacterial surface molecules: Comparison with commensals and pathogens. Nat. Rev. Microbiol. 8, 171–184. doi: 10.1038/nrmicro2297, PMID: 20157338

[ref49] Le Marrec C. (2011). “Responses of lactic acid bacteria to osmotic stress” in Stress Responses of Lactic Acid Bacteria. eds. Tsakalidou E. Papadimitriou K. (Boston, MA: Food Microbiology and Food Safety. Springer).

[ref50] Le Marrec C. Bon E. Lonvaud-Funel A. (2007). Tolerance to high osmolality of the lactic acid bacterium *Oenococcus oeni* and identification of potential osmoprotectants. Int. J. Food Microbiol. 115, 335–342. doi: 10.1016/j.ijfoodmicro.2006.12.039, PMID: 17320992

[ref51] Le D. T. Tran T. L. Duviau M. P. Meyrand M. Guérardel Y. Castelain M.. (2013). Unraveling the role of surface mucus-binding protein and pili in muco-adhesion of *Lactococcus lactis*. PLoS One 8:e79850. doi: 10.1371/journal.pone.0079850, PMID: 24260308 PMC3832589

[ref52] LeBlanc J. G. Chain F. Martín R. Bermúdez-Humarán L. G. Courau S. Langella P. (2017). Beneficial effects on host energy metabolism of short-chain fatty acids and vitamins produced by commensal and probiotic bacteria. Microb. Cell Factories 16:79. doi: 10.1186/s12934-017-0691-z, PMID: 28482838 PMC5423028

[ref53] LeBlanc J. G. del Carmen S. Miyoshi A. (2011). Use of superoxide dismutase and catalase producing lactic acid bacteria in TNBS induced Crohn's disease in mice. J. Biotechnol. 151, 287–293. doi: 10.1016/j.jbiotec.2010.11.008, PMID: 21167883

[ref54] Lee I. C. Tomita S. Kleerebezem M. Bron P. A. (2013). The quest for probiotic effector molecules—unraveling strain specificity at the molecular level. Pharmacol. Res. 69, 61–74. doi: 10.1016/j.phrs.2012.09.010 23059538

[ref55] Lippolis R. Siciliano R. A. Mazzeo M. F. Abbrescia A. Gnoni A. Sardanelli A. M.. (2013). Comparative secretome analysis of four isogenic *Bacillus clausii* probiotic strains. Proteome Sci. 11:28. doi: 10.1186/1477-5956-11-28, PMID: 23816335 PMC3716886

[ref56] Louis P. Flint H. J. (2017). Formation of propionate and butyrate by the human colonic microbiota. Environ. Microbiol. 19, 29–41. doi: 10.1111/1462-2920.13589 27928878

[ref57] Ma X. Wang G. Zhai Z. Zhou P. Hao Y. (2018). Global transcriptomic analysis and function identification of malolactic enzyme pathway of *Lactobacillus paracasei* L9 in response to bile stress. Front. Microbiol. 9:1978. doi: 10.3389/fmicb.2018.01978, PMID: 30210466 PMC6119781

[ref58] Mager L. F. Wasmer M. H. Rau T. T. Krebs P. (2016). Cytokine-induced modulation of colorectal cancer. Front. Oncol. 6:96. doi: 10.3389/fonc.2016.00096, PMID: 27148488 PMC4835502

[ref59] Malberg Tetzschner A. M. Johnson J. R. Johnston B. D. Lund O. Scheutz F. (2020). *In silico* genotyping of *Escherichia coli* isolates for extraintestinal virulence genes by use of whole-genome sequencing data. J. Clin. Microbiol. 58, e01269–e01320. doi: 10.1128/JCM.01269-20, PMID: 32669379 PMC7512150

[ref60] Mantzourani I. Chondrou P. Bontsidis C. Karolidou K. Terpou A. Alexopoulos A.. (2019). Assessment of the probiotic potential of lactic acid bacteria isolated from kefir grains: evaluation of adhesion and antiproliferative properties in in vitro experimental systems. Ann. Microbiol. 69, 751–763. doi: 10.1007/s13213-019-01467-6

[ref61] Markowiak P. Śliżewska K. (2017). Effects of probiotics, prebiotics, and synbiotics on human health. Nutrients 9:1021. doi: 10.3390/nu9091021, PMID: 28914794 PMC5622781

[ref62] Martinez V. Ingwers M. Smith J. Glushka J. Yang T. Bar-Peled M. (2012). Biosynthesis of UDP-4-keto-6-deoxyglucose and UDP-rhamnose in pathogenic fungi *Magnaporthe grisea* and *Botryotinia fuckeliana*. J. Biol. Chem. 287, 879–892. doi: 10.1074/jbc.M111.287367, PMID: 22102281 PMC3256918

[ref63] Matsuzaki T. Chin J. (2000). Modulating immune responses with probiotic bacteria. Immunol. Cell Biol. 78, 67–73. doi: 10.1046/j.1440-1711.2000.00887.x 10651931

[ref64] Meyrand M. Guillot A. Goin M. Furlan S. Armalyte J. Kulakauskas S.. (2013). Surface proteome analysis of a natural isolate of *Lactococcus lactis* reveals the presence of pili able to bind human intestinal epithelial cells. Mol. Cell. Proteomics 12, 3935–3947. doi: 10.1074/mcp.M113.029066, PMID: 24002364 PMC3861735

[ref65] Mistou M.-Y. Sutcliffe I. C. van Sorge N. M. (2016). Bacterial glycobiology: rhamnose-containing cell wall polysaccharides in gram-positive bacteria. FEMS Microbiol. Rev. 40, 464–479. doi: 10.1093/femsre/fuw006, PMID: 26975195 PMC4931226

[ref66] Mistry J. Chuguransky S. Williams L. (2021). Pfam: the protein families database in 2021. Narrative 49, D412–D419. doi: 10.1093/nar/gkaa913, PMID: 33125078 PMC7779014

[ref67] Muscariello L. De Siena B. Marasco R. (2020). *Lactobacillus* cell surface proteins involved in interaction with mucus and extracellular matrix components. Curr. Microbiol. 77, 3831–3841. doi: 10.1007/s00284-020-02243-5, PMID: 33079206 PMC7677277

[ref68] Nami Y. Abdullah N. Haghshenas B. Radiah D. Rosli R. Khosroushahi A. Y. (2014a). Probiotic potential and biotherapeutic effects of newly isolated vaginal *Lactobacillus acidophilus* 36YL strain on cancer cells. Anaerobe 28, 29–36. doi: 10.1016/j.anaerobe.2014.04.012, PMID: 24818631

[ref69] Nami Y. Abdullah N. Haghshenas B. Radiah D. Rosli R. Yari Khosroushahi A. (2014b). A newly isolated probiotic *Enterococcus faecalis* strain from vagina microbiota enhances apoptosis of human cancer cells. J. Appl. Microbiol. 117, 498–508. doi: 10.1111/jam.12531, PMID: 24775273

[ref70] Nishikawa M. Tamada A. Hyoudou K. Umeyama Y. Takahashi Y. Kobayashi Y.. (2004). Inhibition of experimental hepatic metastasis by targeted delivery of catalase in mice. Clin. Exp. Metastasis 21, 213–221. doi: 10.1023/b:clin.0000037706.13747.5e, PMID: 15387371

[ref71] Nobaek S. Johansson M. L. Molin G. Ahrné S. Jeppsson B. (2000). Alteration of intestinal microflora is associated with reduction in abdominal bloating and pain in patients with irritable bowel syndrome. Am. J. Gastroenterol. 95, 1231–1238. doi: 10.1111/j.1572-0241.2000.02015.x, PMID: 10811333

[ref72] Nwodo U. U. Green E. Okoh A. I. (2012). Bacterial exopolysaccharides: functionality and prospects. Int. J. Mol. Sci. 13, 14002–14015. doi: 10.3390/ijms131114002, PMID: 23203046 PMC3509562

[ref73] Obis D. Guillot A. Gripon J. C. Renault P. Bolotin A. Mistou M. Y. (1999). Genetic and biochemical characterization of a high-affinity betaine uptake system (BusA) in *Lactococcus lactis* reveals a new functional organization within bacterial ABC transporters. J. Bacteriol. 181, 6238–6246. doi: 10.1128/JB.181.20.6238-6246.1999, PMID: 10515910 PMC103755

[ref74] Obis D. Guillot A. Mistou M. Y. (2001). Tolerance to high osmolality of *Lactococcus lactis* subsp. *lactis* and *cremoris* is related to the activity of a betaine transport system. FEMS Microbiol. Lett. 202, 39–44. doi: 10.1111/j.1574-6968.2001.tb10777.x, PMID: 11506905

[ref75] Oliveira L. C. Saraiva T. D. L. Silva W. M. Pereira U. P. Campos B. C. Benevides L. J.. (2017). Analyses of the probiotic property and stress resistance-related genes of *Lactococcus lactis* subsp. *lactis* NCDO 2118 through comparative genomics and in vitro assays. PLoS One 12:e0175116. doi: 10.1371/journal.pone.0175116, PMID: 28384209 PMC5383145

[ref76] Op den Camp H. J. Oosterhof A. Veerkamp J. H. (1985). Interaction of bifidobacterial lipoteichoic acid with human intestinal epithelial cells. Infect. Immun. 47, 332–334. doi: 10.1128/iai.47.1.332-334.1985, PMID: 3965407 PMC261519

[ref77] Orlando A. Refolo M. G. Messa C. Amati L. Lavermicocca P. Guerra V.. (2012). Antiproliferative and proapoptotic effects of viable or heat-killed *Lactobacillus paracasei* IMPC2.1 and *Lactobacillus rhamnosus* GG in HGC-27 gastric and DLD-1 colon cell lines. Nutr. Cancer 64, 1103–1111. doi: 10.1080/01635581.2012.717676, PMID: 23061912

[ref78] Pala V. Sieri S. Berrino F. Vineis P. Sacerdote C. Palli D.. (2011). Yogurt consumption and risk of colorectal cancer in the Italian European prospective investigation into cancer and nutrition cohort. Int. J. Cancer 129, 2712–2719. doi: 10.1002/ijc.26193, PMID: 21607947

[ref79] Piuri M. Sanchez-Rivas C. Ruzal S. M. (2003). Adaptation to high salt in *Lactobacillus*: role of peptides and proteolytic enzymes. J. Appl. Microbiol. 95, 372–379. doi: 10.1046/j.1365-2672.2003.01971.x, PMID: 12859771

[ref80] Potter S. C. Luciani A. Eddy S. R. Park Y. Lopez R. Finn R. D. (2018). HMMER web server: 2018 update. Narrative 46, W200–W204. doi: 10.1093/nar/gky448, PMID: 29905871 PMC6030962

[ref81] Prete R. Long S. L. Gallardo A. L. Gahan C. G. Corsetti A. Joyce S. A. (2020). Beneficial bile acid metabolism from *Lactobacillus plantarum* of food origin. Sci. Rep. 10:1165. doi: 10.1038/s41598-020-58069-5, PMID: 31980710 PMC6981223

[ref82] Radziwiłł-Bieńkowska J. Le D. T. Szczęsny P. Duviau M. P. Aleksandrzak-Piekarczyk T. Loubière P.. (2016). Adhesion of the genome-sequenced *Lactococcus lactis* subsp. *cremoris* IBB477 strain is mediated by specific molecular determinants. J. Appl. Microbiol. Biotechnol. 100, 9605–9617. doi: 10.1007/s00253-016-7813-0, PMID: 27687992 PMC5071367

[ref83] Radziwiłł-Bieńkowska J. M. Robert V. Drabot K. Chain F. Cherbuy C. Langella P.. (2017). Contribution of plasmid-encoded peptidase S8 (PrtP) to adhesion and transit in the gut of *Lactococcus lactis* IBB477 strain. Appl. Microbiol. Biotechnol. 101, 5709–5721. doi: 10.1007/s00253-017-8334-1, PMID: 28540425 PMC5501904

[ref84] Reid G. Bocking A. (2003). The potential for probiotics to prevent bacterial vaginosis and preterm labor. Am. J. Obstet. Gynecol. 189, 1202–1208. doi: 10.1067/s0002-9378(03)00495-2, PMID: 14586379

[ref85] Rekha C. R. Vijayalakshmi G. (2010). Bioconversion of isoflavone glycosides to aglycones, mineral bioavailability and vitamin B complex in fermented soymilk by probiotic bacteria and yeast. J. Appl. Microbiol. 109, 1198–1208. doi: 10.1111/j.1365-2672.2010.04745.x, PMID: 20477889

[ref86] Robert H. Le Marrec C. Blanco C. Jebbar M. (2000). Glycine betaine, carnitine, and choline enhance salinity tolerance and prevent the accumulation of sodium to a level inhibiting growth of *Tetragenococcus halophila*. Appl. Environ. Microbiol. 66, 509–517. doi: 10.1128/AEM.66.2.509-517.2000, PMID: 10653711 PMC91856

[ref87] Salminen S. Nurmi J. Gueimonde M. (2005). The genomics of probiotic intestinal microorganisms. Genome Biol. 6:225. doi: 10.1186/gb-2005-6-7-225xx, PMID: 15998456 PMC1175979

[ref88] Saxelin M. Tynkkynen S. Mattila-Sandholm T. de Vos W. M. (2005). Probiotic and other functional microbes: from markets to mechanisms. Curr. Opin. Biotechnol. 16, 204–211. doi: 10.1016/j.copbio.2005.02.003, PMID: 15831388

[ref89] Serban D. E. (2014). Gastrointestinal cancers: influence of gut microbiota, probiotics and prebiotics. Cancer Lett. 345, 258–270. doi: 10.1016/j.canlet.2013.08.013 23981580

[ref91] Settanni C. R. Quaranta G. Bibbò S. Gasbarrini A. Cammarota G. Ianiro G. (2020). Oral supplementation with lactobacilli to prevent colorectal cancer in preclinical models. Minerva Gastroenterol. Dietol. 66, 48–69. doi: 10.23736/S1121-421X.19.02631-X, PMID: 31760735

[ref92] Sniffen J. C. McFarland L. V. Evans C. T. Goldstein E. J. C. (2018). Choosing an appropriate probiotic product for your patient: an evidence-based practical guide. PLoS One 13:e0209205. doi: 10.1371/journal.pone.0209205, PMID: 30586435 PMC6306248

[ref93] Tannock G. W. Dashkevitz M. P. Feighner S. D. (1989). Lactobacilli and bile salt hydrolase in the murine intestinal tract. Appl. Environ. Microbiol. 55, 1848–1851. doi: 10.1128/aem.55.7.1848-1851.1989, PMID: 2527484 PMC202961

[ref94] Taverniti V. Guglielmetti S. (2011). The immunomodulatory properties of probiotic microorganisms beyond their viability (ghost probiotics: proposal of paraprobiotic concept). Genes Nutr. 6, 261–274. doi: 10.1007/s12263-011-0218-x, PMID: 21499799 PMC3145061

[ref95] Thirabunyanon M. Hongwittayakorn P. (2013). Potential probiotic lactic acid bacteria of human origin induce antiproliferation of colon cancer cells via synergic actions in adhesion to cancer cells and short-chain fatty acid bioproduction. Appl. Biochem. Biotechnol. 169, 511–525. doi: 10.1007/s12010-012-9995-y, PMID: 23239414

[ref96] Turpin W. Humblot C. Noordine M.-L. Thomas M. Guyot J.-P. (2012). *Lactobacillaceae* and cell adhesion: genomic and functional screening. PLoS One 7:e38034. doi: 10.1371/journal.pone.0038034, PMID: 22675431 PMC3364998

[ref97] Uguen P. Hamelin J. Le Pennec J. P. Blanco C. (1999). Influence of osmolarity and the presence of an osmoprotectant on *Lactococcus lactis* growth and bacteriocin production. Appl. Environ. Microbiol. 65, 291–293. doi: 10.1128/AEM.65.1.291-293.1999, PMID: 9872793 PMC91016

[ref98] Vamanu E. Gatea F. (2020). Correlations between microbiota bioactivity and bioavailability of functional compounds: a mini-review. Biomedicine 8:39. doi: 10.3390/biomedicines8020039, PMID: 32093399 PMC7167868

[ref99] Ventura M. Turroni F. van Sinderen D. (2012). Probiogenomics as a tool to obtain genetic insights into adaptation of probiotic bacteria to the human gut. Bioeng. Bugs 3, 73–79. doi: 10.4161/bbug.18540, PMID: 22095053 PMC3357336

[ref100] Waldecker M. Kautenburger T. Daumann H. Veeriah S. Will F. Dietrich H.. (2008). Histone-deacetylase inhibition and butyrate formation: fecal slurry incubations with apple pectin and apple juice extracts. Nutrition 24, 366–374. doi: 10.1016/j.nut.2007.12.013, PMID: 18262392

[ref101] Wang G. Yu Y. Garcia-Gutierrez E. Jin X. He Y. Wang L.. (2019). *Lactobacillus acidophilus* JCM 1132 strain and its mutant with different bacteriocin-producing behaviour have various in situ effects on the gut microbiota of healthy mice. Microorganisms 8:49. doi: 10.3390/microorganisms8010049, PMID: 31881756 PMC7022661

[ref102] Wick R. R. Judd L. M. Gorrie C. L. Holt K. E. (2017). Unicycler: resolving bacterial genome assemblies from short and long sequencing reads. PLoS Comput. Biol. 13:e1005595. doi: 10.1371/journal.pcbi.1005595, PMID: 28594827 PMC5481147

[ref103] Wu J. Zhang Y. Ye L. Wang C. (2021). The anti-cancer effects and mechanisms of lactic acid bacteria exopolysaccharides *in vitro*: a review. Carbohydr. Polym. 253:117308. doi: 10.1016/j.carbpol.2020.117308, PMID: 33278957

[ref104] Yao W. Yang L. Shao Z. Xie L. Chen L. (2020). Identification of salt tolerance-related genes of *Lactobacillus plantarum* D31 and T9 strains by genomic analysis. Ann. Microbiol. 70:10. doi: 10.1186/s13213-020-01551-2

[ref105] Yoshii K. Hosomi K. Sawane K. Kunisawa J. (2019). Metabolism of dietary and microbial vitamin b family in the regulation of host immunity. Front. Nutr. 6:48. doi: 10.3389/fnut.2019.00048, PMID: 31058161 PMC6478888

[ref106] Yu N. Y. Wagner J. R. Laird M. R. Melli G. Rey S. Lo R.. (2010). PSORTb 3.0: improved protein subcellular localization prediction with refined localization subcategories and predictive capabilities for all prokaryotes. Bioinformatics 26, 1608–1615. doi: 10.1093/bioinformatics/btq249, PMID: 20472543 PMC2887053

[ref107] Zhang K. Dai H. Liang W. Zhang L. Deng Z. (2019). Fermented dairy foods intake and risk of cancer. Int. J. Cancer 144, 2099–2108. doi: 10.1002/ijc.31959, PMID: 30374967

[ref108] Zhao C. Dong H. Zhang Y. Li Y. (2019). Discovery of potential genes contributing to the biosynthesis of short-chain fatty acids and lactate in gut microbiota from systematic investigation in *E. coli*. NPJ Biofilms Microbiomes 5:19. doi: 10.1038/s41522-019-0092-7, PMID: 31312512 PMC6626047

[ref109] Zhong L. Zhang X. Covasa M. (2014). Emerging roles of lactic acid bacteria in protection against colorectal cancer. World J. Gastroenterol. 20, 7878–7886. doi: 10.3748/wjg.v20.i24.7878, PMID: 24976724 PMC4069315

[ref110] Zhou Y. Liang Y. Lynch K. H. Dennis J. J. Wishart D. S. (2011). PHAST: a fast phage search tool. Narrative 39, W347–W352. doi: 10.1093/nar/gkr485, PMID: 21672955 PMC3125810

[ref111] Zielińska D. Kolożyn-Krajewska D. (2018). Food-origin lactic acid bacteria may exhibit probiotic properties: review. Biomed. Res. Int. 2018:5063185. doi: 10.1155/2018/5063185, PMID: 30402482 PMC6191956

[ref112] Zitvogel L. Daillère R. Roberti M. P. Routy B. Kroemer G. (2017). Anticancer effects of the microbiome and its products. Nat. Rev. Microbiol. 15, 465–478. doi: 10.1038/nrmicro.2017.44 28529325

